# Photochemical Fuel Carrier Molecules for Robotic Embodied Energy

**DOI:** 10.1002/adma.202520447

**Published:** 2026-02-28

**Authors:** Chuqi Huang, Songah Jeong, Ji Woo Kim, Kanyarat Mantala, Zenghao Zhang, Hyungwoo Kim, Abdon Pena‐Francesch

**Affiliations:** ^1^ Department of Materials Science and Engineering University of Michigan Ann Arbor Michigan USA; ^2^ School of Polymer Science and Engineering Chonnam National University Gwangju South Korea; ^3^ Department of Chemical Engineering University of Michigan Ann Arbor Michigan USA; ^4^ Macromolecular Science and Engineering University of Michigan Ann Arbor Michigan USA; ^5^ Robotics Institute University of Michigan Ann Arbor Michigan USA; ^6^ Biointerfaces Institute University of Michigan Ann Arbor Michigan USA

**Keywords:** active matter, embodied energy, hexafluoroisopropanol, microrobotics, *o*‐nitrobenzyl, photoresponsive molecules, self‐immolative, surface tension

## Abstract

The downsizing of mobile robots faces obstacles in power and control as conventional electromechanical systems do not scale favorably with mass and size. Despite recent advancements in functional materials, enabling efficient energy storage, structural integration, and on‐demand energy release in small‐scale robots remains a challenge. Inspired by metabolic strategies in animals, we designed a fuel carrier molecule for embodied energy in small‐scale swimming robots. We integrated an ultralow surface tension unit into a photolabile *o*‐nitrobenzyl derivative to yield a novel photoresponsive molecule for fuel storage and controlled release. Combining these two properties, carrier molecules undergo bond scission under UV light to release fuel and locally manipulate surface tension to generate Marangoni flows. We incorporated this fuel carrier into an easily processable polymer composite to enable its application as a structural component with embodied energy and control. We implemented this approach in Marangoni micropump systems, surface‐tension‐active particle transportation, and untethered hybrid microrobots that combine photochemical on–off propulsion with magnetic control. This materials platform serves as a versatile solution to store fuel and energy in structural components and release it on demand with precision, opening new opportunities for embodied energy design in microrobots, soft devices, and active matter systems.

## Graphical abstract

We designed a novel fuel carrier photoresponsive molecule for on‐demand energy storage and release. Carrier molecules undergo bond scission under UV light to release chemical fuel and locally generate propulsive Marangoni flows. We demonstrate this photochemical embodied energy strategy in small‐scale robots and active matter systems, enabling micropumps, particle manipulation, and untethered microrobot control.

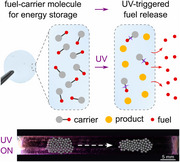

## 1 Introduction

Small‐scale mobile robots [1] are increasingly being recognized as a versatile technology suitable for a wide range of applications in medicine [2, 3], manufacturing [4, 5], environmental remediation [6, 7], and exploration, particularly where access to confined and unstructured spaces is needed. As robot sizes are scaled down, conventional electromechanical parts are impractical and difficult to integrate into miniaturized designs due to inherent space and weight constraints. Researchers have been actively exploring novel alternative power and locomotion methods for these small‐scale robots, including the use of microelectromechanical actuators [8], magnetic fields [9, 10], acoustic waves [11, 12], light [13, 14], and chemical reactions [15, 16]. Among these, chemically‐driven robots offer the advantage of integrating diverse on‐board fuels and energy‐dense materials (in the form of a chemical reservoir or multifunctional structural component) to provide untethered autonomy [17]. This strategy is broadly used in the animal kingdom through metabolism, where food is converted into a usable form of energy and stored in the body to be later consumed on demand. Other examples of chemically‐driven processes in animal locomotion include the direct release of fuel biomolecules to induce fast evasive propulsion in insects [18]. For instance, water treaders (*Mesovelia* and *Microvelia*) are semiaquatic insects that can quickly evade predators by locally secreting surfactant biomolecules to reduce the surface tension and generate Marangoni propulsive forces [19]. This surface tension propulsion mechanism has inspired many aquatic robot designs, ranging from chemical reservoirs with mechanical valves to fuel‐embedded micro/nanoporous materials [5, 20, 21, 22, 23]. Passive mechanisms lack control over the fuel release rate (constant release), while active mechanisms usually result in larger robot footprint (which in turn increases weight and hydrodynamic drag). Thus, efficient power, flexible control, and long operational lifetime remain major barriers in the miniaturization, design, and operation of untethered chemically‐powered small‐scale robots.

Molecular design of chemical fuels for small‐scale robots offers novel opportunities not only to regulate fuel release efficiently, but also to give rise to versatile control functions. In this regard, developing stimuli‐responsive molecules that become active upon exposure to external triggers (e.g., light, heat, pH, chemical, and humidity) enables precise control over microrobotic systems, thereby inducing macroscopic responses such as large‐scale motion, shape/volume change, and controlled propulsion [24]. Among these stimuli, light has been extensively employed to impart spatiotemporal control owing to its high precision, rapidity, and accessibility [13, 25], often through light‐responsive moieties, such as nitrobenzyl, coumarin, azobenzene, and spiropyran derivatives, in combination with synergistic additives [26, 27, 28, 29, 30]. In parallel, from the perspective of reaction mechanism engineering, self‐immolative chemistry, characterized by predictable covalent bond‐cleavage cascades with high efficiency and fidelity, has emerged as a particular notable strategy because of its unique ability to transform fleeting and/or trace stimuli into amplified signals [31, 32]. Thus, numerous self‐immolative polymeric materials have been developed to achieve controlled degradation, triggered release, and monomer recycling [33, 34, 35, 36]. Nonetheless, such self‐immolative mechanisms have been only limitedly applied to degradable robotic systems [37, 38, 39], and seldom explored for the molecular design of responsive fuels. Therefore, we aim to design a self‐immolative fuel carrier that releases a target fuel molecule in response to light. This approach not only alleviates typical drawbacks of small‐mass fuels, such as leakage, inefficiency, poor controllability, and low bench‐top stability, but also enables on‐demand, molecular‐level control for the soft robots.

Here, we have designed a carrier molecule system to incorporate chemical fuel in a stable form for storage, and later release it on demand upon photolysis. We identified hexafluoro isopropanol (HFIP) [40] as an ideal fuel molecule for surface tension propulsion due to several key properties: (i) soluble in water, (ii) very low surface tension (γ_HFIP_ = 14.7 mN m^−1^) even at extremely low concentrations, (iii) permeable to UV light, (iv) highly volatile (will not accumulate and saturate the liquid media), and (v) low toxicity (used in anesthetics) [22, 41]. However, due to its low molecular weight and low boiling point, HFIP fuel is not stable when incorporated directly in a Marangoni motor system as it can quickly evaporate and/or continuously leak into the liquid media (thus reducing its lifetime and preventing storage). We have incorporated HFIP into a photocleavable carrier molecule, an *o*‐nitrobenzyl (ONB) derivative, that can be safely stored without degradation or loss of fuel and can be easily processed into different composite materials and form factors. When fuel is needed, on‐demand exposure to UV light triggers the controlled photolysis of the carrier molecule generating in situ HFIP fuel (to be used for propulsion) and a scission byproduct (that will remain trapped in the motor). Leveraging this novel molecular design of photocleavable precursors, we have integrated chemical fuel in stable carrier form in a polymer composite system, readily available for use within the material and structure for long storage and operation times, which can be then converted in situ into usable fuel as needed via photolysis. We demonstrate the properties and applications of this photolytic fuel carrier in a Marangoni micropump system, surface tension active particles, and hybrid microrobots with controlled propulsion (on demand go/stop functions) and trajectories (magnetic steering).

## 2 Results and Discussion

### 2.1 Design of Photolytic Fuel Carrier Molecules

**FIGURE 1:**
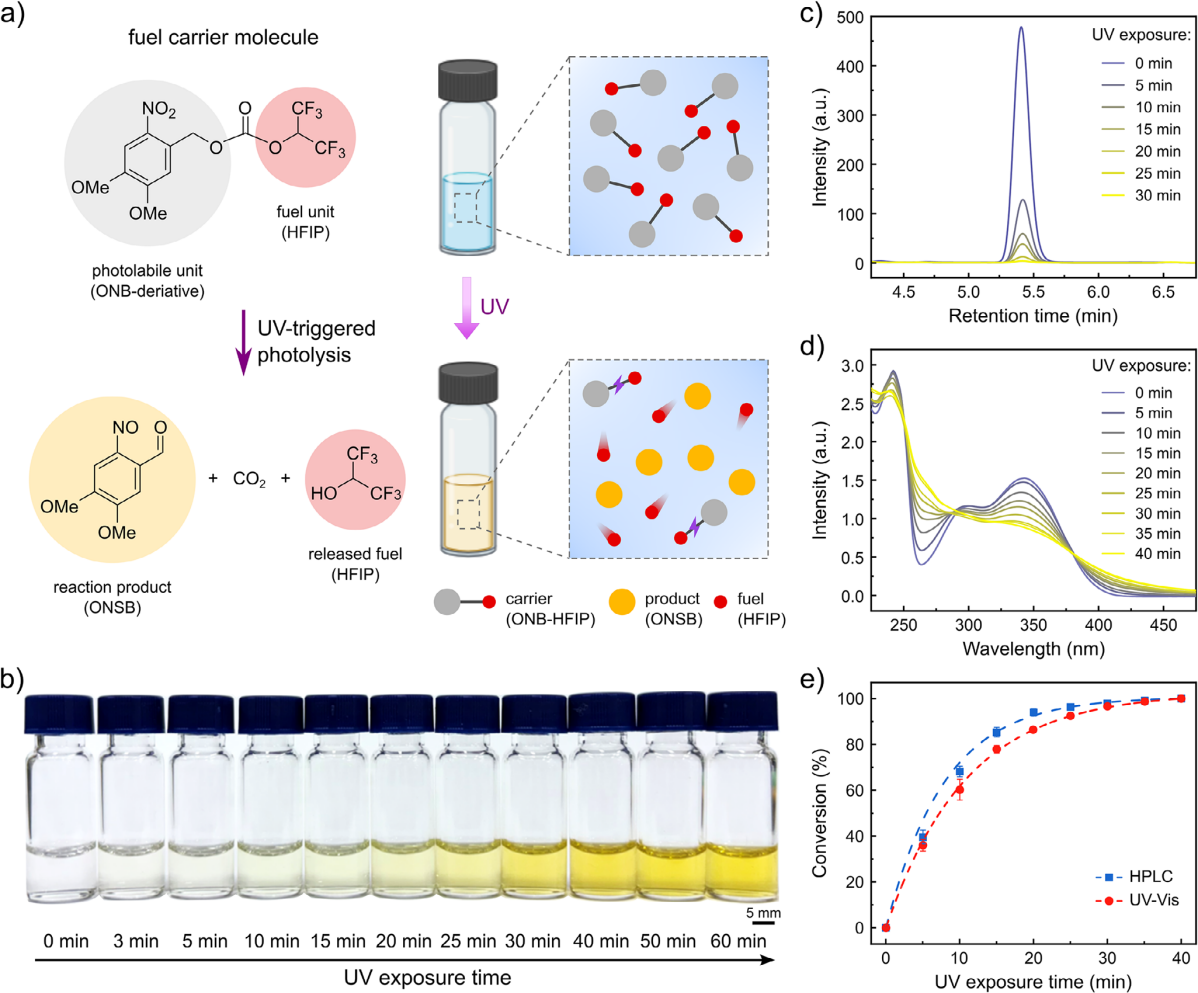
Light‐triggered chemical fuel generation from photolytic fuel‐carrier molecules. (a) Design and photolytic mechanism of fuel‐carrier molecules, ONB‐HFIP, which undergo UV‐triggered photolysis to release HFIP fuel and reaction byproducts. (b) Color change of ONB‐HFIP in solution as a function of UV irradiation time. (c) Time‐resolved HPLC chromatograms and (d) UV‐vis absorption spectra of ONB‐HFIP in solution as a function of UV irradiation time. (e) Conversion degree of photolytic reaction, estimated from the changing ratio of HPLC peak areas and UV‐vis absorption intensities at 343 and 264 nm over time.

We have designed a light‐responsive carrier molecule that is capable of releasing HFIP fuel in response to UV‐light irradiation (Figure 1a). The carrier molecule (ONB‐HFIP) could be readily synthesized by introducing a 4,5‐dimethoxy‐2‐nitrobenzyl carbonate (*o*‐nitrobenzyl; ONB unit) onto HFIP (chemical fuel unit) via a nucleophilic acyl substitution reaction in a high yield. We selected the ONB unit specifically because (i) it is more hydrophobic than HFIP (and therefore not water soluble) and (ii) it exhibits selective and rapid photolabile capability under UV irradiation while liberating CO_2_ [42, 43]. Based on its molecular weight, 41% of the ONB‐HFIP fuel carrier molecule contains usable fuel, 48% remains as a reaction byproduct, and 11% is lost as released CO_2_. These characteristics enabled not only loading of the thermally stable carrier molecule into various polymer matrices without loss of fuel but also the light‐triggered uncaging of HFIP fuel in a readily usable form with spatiotemporal precision under aqueous conditions. The chemical structure of ONB‐HFIP was thoroughly characterized by nuclear magnetic resonance (NMR) and Fourier transform infrared (FTIR) spectroscopy (Figures S1‐S3), confirming the presence of both ONB and trifluoromethyl moieties.

The light‐responsive behavior of the ONB‐HFIP carrier molecule was investigated in solution, prior to use. Upon 365‐nm irradiation (3 mW cm^−2^), the initially transparent solution of ONB‐HFIP (2 mM in MeCN) gradually turned yellow (Figure 1b), which could be attributed to the formation of the photochemical products, including *o*‐nitrosobenzaldehyde (ONSB) [42]. The visual color change was estimated using the CMYK color system, in which the yellow (Y) value increased from 0 to 70 during UV exposure and approached saturation after 30 min (Figure S4). The photocleavage reaction of ONB‐HFIP in solution was further analyzed using high‐performance liquid chromatography (HPLC) and UV‐vis spectroscopy. Under irradiation with 365 nm light, time‐dependent disappearance of the ONB‐HFIP peak was observed in the elution profile with 8:2 MeCN–H_2_O (v/v) (Figure 1c). The initial peak that appeared at 5.4 min diminished to negligible levels after 30‐min irradiation, supporting complete photocleavage of the carrier ONB‐HFIP molecule. In addition, ONB‐HFIP exhibited a broad absorption peak around 343 nm (Figure 1d), which progressively decreased upon UV exposure, accompanied by an increase in absorption near 264 nm and above 381 nm. This UV‐vis spectral change, along with two isosbestic points at 288 and 381 nm, indicates the occurrence of a photochemical reaction of the carrier, leading to the generation of the nitrosobenzene products and the concomitant release of HFIP fuel. The rate of consumption of ONB‐HFIP for each case was evaluated as shown in Figure 1e, by comparing the time‐lapse changes in the HPLC peak area and the UV‐vis absorbance intensity ratio at 343 and 264 nm (I_343_/I_264_). Both consistently indicated that the conversion was almost completed and reached saturation after 30 min. When estimated using a pseudo‐first‐order kinetic model, the reaction rate (*k*) is ∼0.11 min^−1^ for HPLC and ∼0.07 min^−1^ for UV‐vis. For comparison, control experiments under incandescent light instead of UV irradiation showed no significant conversion (Figure S5).

### 2.2 Integration of Fuel Carrier Molecules within a Functional Polymer Composite

In solution, the ONB‐HFIP photocleavage reaction proceeded efficiently under UV irradiation. However, in the solid state, the photocleavage was barely detectable. Figure S6 presents the ^1^H NMR and FTIR spectra of the carrier molecule before and after UV exposure as dry powders. Both characterization results confirm that negligible photocleavage occurred under these conditions. It has been reported that the photolytic efficiency of ONB moieties is highly related with its physical state, which is usually lower in the solid state than in a liquid medium because of difficulty in attaining appropriate conformations for photoreaction, limited light penetration depth, and lower specific surface area in the solid state [42, 44, 45, 46].

**FIGURE 2:**
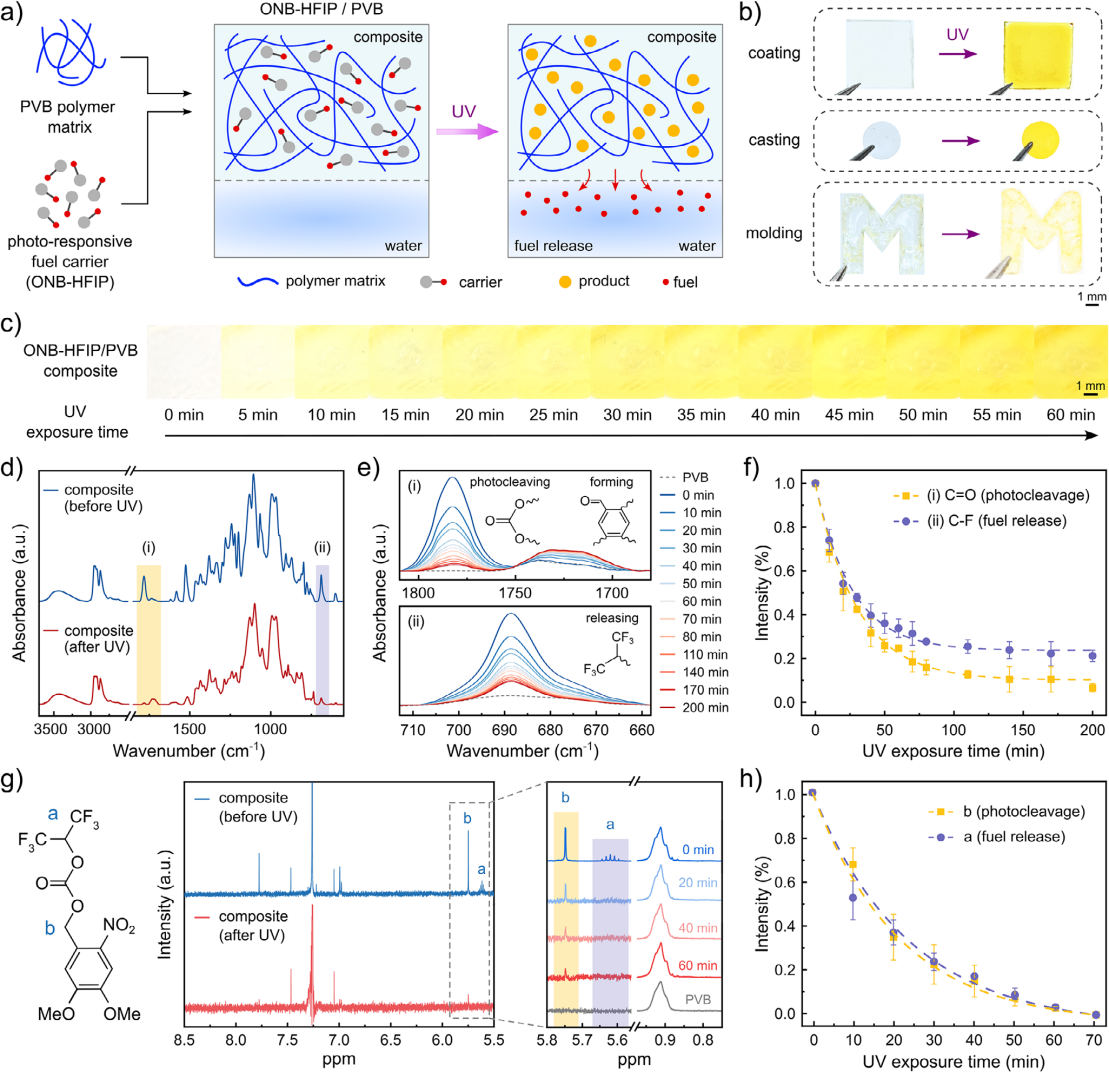
Integration of fuel‐carrier molecules within a composite system. (a) Fabrication and photo‐triggered fuel generation of ONB‐HFIP/PVB composites, with ONB‐HFIP molecules dispersed in a PVB polymer matrix, and to generate and release HFIP as chemical fuel upon UV via ONB's photolytic reaction. (b) Processing of ONB‐HFIP/PVB composites, demonstrated through coating, casting, and molding. (c) Color change of ONB‐HFIP/PVB composites as a function of different UV irradiation time. (d) Infrared spectra of composite systems before and after UV irradiation. (e) Evolution of FTIR peaks of (i) C═O stretching in carbonate group and benzaldehyde group, and (ii) C─F symmetric deformation in trifluoromethyl group reveal photocleavage, byproduct formation, and chemical fuel release, respectively; peaks from PVB were used as a control here. (f) Time‐resolved changes in photocleavage and fuel release, estimated by changes in FTIR peak areas of (i) and (ii), respectively. (g) Chemical structure of ONB‐HFIP (left), NMR spectra of ONB‐HFIP/PVB composite before and after UV irradiation (middle), and magnified view of *a* and *b* peaks comparing with the peak from butyral units in PVB (right). h) NMR peak areas corresponding to fuel release (*a* peak) and cleavage reaction (*b* peak) over UV irradiation time.

To overcome the intrinsic limitations of ONB‐based powders and expand the applicability of our system, we designed a conformal and modular coating system by embedding ONB‐HFIP carriers into a poly(vinyl butyral) (PVB) matrix (Figure 2a). Herein, PVB offers several advantages: (i) its disordered backbone provides a flexible, adhesive, tough binder for ONB‐HFIP carriers with good dispersibility, (ii) its amorphous matrix promotes conformational change of ONB‐HFIP for photolysis as well as facilitates the diffusion of released HFIP into the surrounding environment, (iii) its optical transparency maximizes UV penetration depth, thereby enhancing photocleavage efficiency, and (iv) its mechanical stability withstands fabrication and manipulation without crumbling and cracking. These features together make PVB an attractive platform for enabling solid‐state photochemistry in our carrier system. In this context, we fabricated a chemical motor composite system (ONB‐HFIP/PVB) at various carrier concentrations. In this stable configuration, ONB‐HFIP/PVB can be processed into films and complex geometries via coating, drop casting, and mold processes (Figure 2b), thus enabling the fabrication of multifunctional monolithic structures capable of producing chemical fuel on demand. The photolysis of ONB‐HFIP carriers within the PVB matrix can be monitored as the color of composite films changes from colorless and transparent to intense yellow with UV irradiation (similar to the reaction observed in solution) (Figure 2c). We have also explored other transparent polymer matrices for ONB‐HFIP encapsulation, including silicones, polyurethanes [47], and polyvinylidene fluoride (Figure S7), all of which exhibited the color change to yellow characteristic of photolysis. PVB also presented excellent optical and mechanical stability, with great durability and abrasion resistance for broad application and long‐time storage (Figure S8). We further characterized the mechanical properties of ONB‐HFIP/PVB composites, with ONB‐HFIP exhibiting a plasticizing effect on the matrix at high concentrations and PVB exhibiting a stiffening effect with UV irradiation (Figure S9).

To verify the successful integration of ONB‐HFIP within PVB, we first performed FTIR spectroscopy (Figure S10). The composite exhibited characteristic absorption peaks from both ONB‐HFIP (1782 cm^−1^ from C═O stretching in the carbonate group, 1525 and 1336 cm^−1^ from asymmetric and symmetric N─O stretching in the nitro group, 1105 and 1195 cm^−1^ for C─F stretching and 688 cm^−1^ for F symmetric deformation in trifluoromethyl group) [48, 49, 50] and PVB (broad band at 3425 cm^−1^ for O─H stretching, 2700–2200 cm^−1^ for CH_3_, CH_2_, and CH stretching, and 1740 cm^−1^ for C═O stretching in ester group) [51, 52], confirming effective dispersion of carrier molecules in the composite system. FTIR spectroscopy also enables time‐resolved tracking of functional groups on the film surface, thus providing insight into the dynamic fuel release profile. Upon UV exposure, a clear reduction was observed between the 1300–1000 cm^−1^ region, which is a fingerprint area of HFIP (stretching vibrations of CF_3_, C─O, and C─C) [50]. This decrease directly indicates HFIP release from the composite film. Additionally, the disappearance of bands at 1782, 1525 and 1336 cm^−1^ (corresponding to C═O stretching in carbonate group and asymmetric/symmetric N─O stretching in nitro group, respectively) and the emergence of a new band at 1715–1730 cm^−1^ (characteristic of C═O stretching in benzaldehyde group), confirm ONB cleavage and byproduct formation (Figure 2d). By immersing in DI water for 30 min (mimicking the aquatic working environment), we further confirmed the stability of photolysis byproducts through their unchanged characteristic peaks (Figure S11). To resolve and quantify these dynamic changes, FTIR spectra were normalized to PVB absorption peaks at the O─H band, and two isolated absorption bands at 1782 cm^−1^ (C═O stretching in carbonate group) and 688 cm^−1^ (C─F symmetric deformation in trifluoromethyl group) were tracked as a function of UV exposure time (at intensity 3–5 mW cm^−2^) (Figure 2e). Monitoring the intensity decay of these two bands allowed us to construct a quantitative kinetic profile of both fuel release and ONB cleavage independently. Time‐resolved analysis followed pseudo‐first‐order kinetics, yielding apparent first‐order rate *k* of ∼0.03 min^−1^ for both fuel release and ONB photocleavage (Figure 2f, Figure S12a). The kinetics of the photolysis process were also characterized and modeled as a function of UV illumination intensity, presenting expected faster photolysis rates with increasing UV power (Figure S13).

To further analyze the photocleavage process of ONB‐HFIP in the PVB matrix, we analyzed the photolysis products by NMR. ONB‐HFIP/PVB films were prepared by co‐dissolution and solution casting, and subjected to solid‐state UV irradiation (3 mW cm^−2^). The irradiated ONB‐HFIP/PVB film was subsequently washed with water to remove photodegradation products and later dissolved in CDCl_3_ for ^1^H NMR analysis (Figure S14). The photochemical reaction of the composite was monitored by tracking the benzylic protons in ONB‐HFIP at 5.75 ppm (peak *a*) and the tertiary hydrogen of HFIP at 5.62 ppm (peak *b*). Both peaks gradually decreased under irradiation and became no longer detectable after 60 min (Figure 2 g). Each peak *a* and *b* represents the bond cleavage in the ONB unit and the following HFIP release, respectively. In contrast, the terminal methyl protons of butyral units in PVB, which appeared at 0.92 ppm, remained unchanged during the reaction (Figure 2g inset). Time‐dependent photocleavage profiles were obtained by comparing the integration ratios of peaks *a* and *b* normalized to the methyl protons from the PVB matrix (internal standard) (Figure 2h). The consumption rate of both peaks also followed pseudo‐first‐order kinetics with *k* of ∼0.04 min^−1^ (Figure S12b), suggesting that the PVB matrix provided a favorable environment for ONB cleavage and fuel release. As a control, the non‐irradiated film retained >98% of the NMR signals even after washing process, which we attribute to the high stability of ONB‐HFIP carrier molecules within the PVB matrix (Figure S15). These results demonstrate that ONB–HFIP/PVB composites enable robust, quantifiable, and tunable photochemistry in the solid state, overcoming the limitations of ONB molecules in solution, while providing high stability to the carrier molecules and enabling their processing into a variety of form factors.

### 2.3 Implementation of Photolytic Composites in Surface Tension Micropumps

**FIGURE 3:**
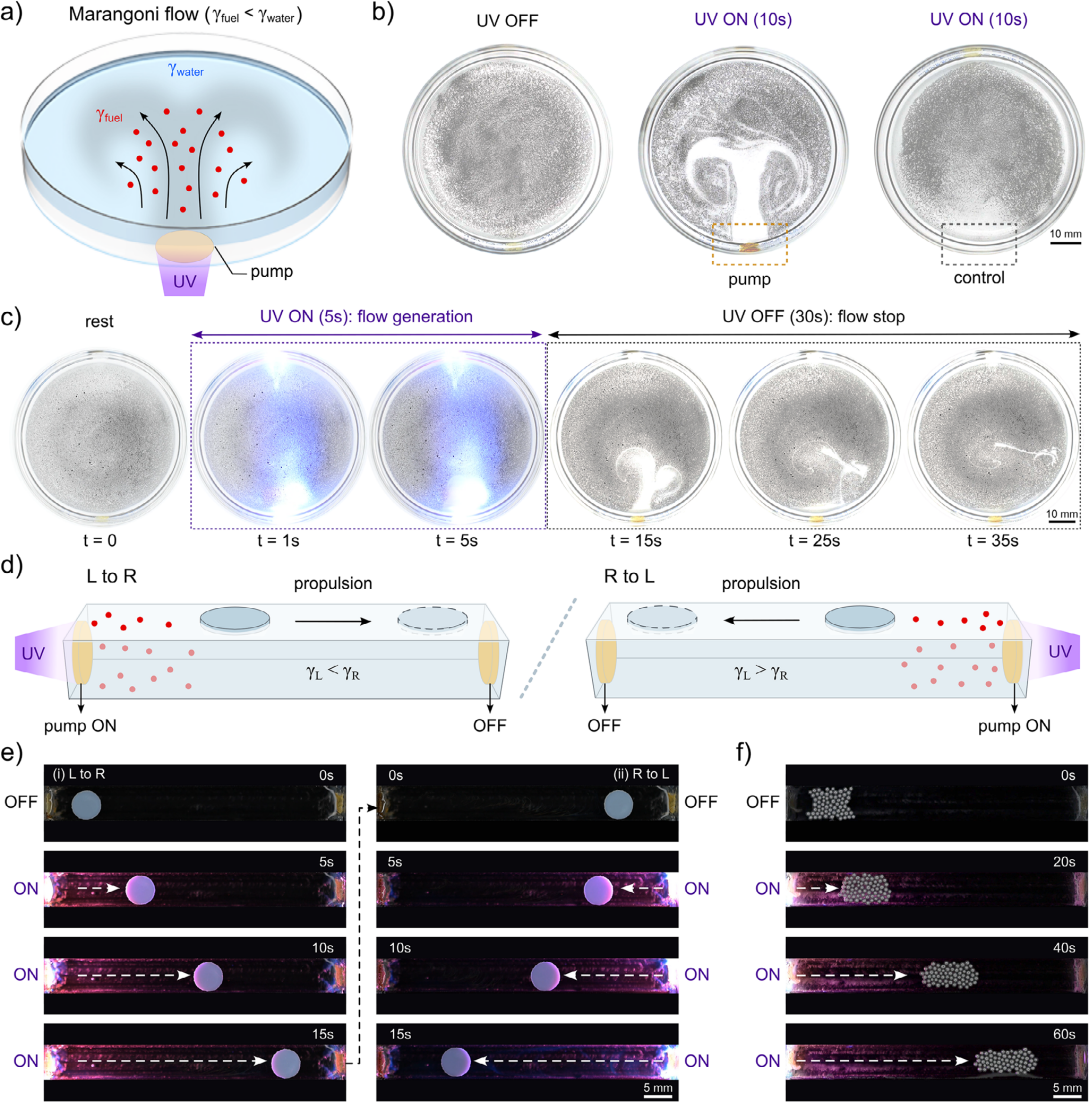
Photochemical micropumps. (a) Application and mechanism of ONB‐HFIP/PVB composites as photo‐responsive chemical pump: fuel releases via photo‐triggered reaction into surrounding water, leading to surface tension gradients and following generation of Marangoni flow. (b) Visualization of flow patterns before and after UV irradiation with pump coating or control (PVB coating without ONB‐HFIP). (c) Cyclic on/off pumping tests, with each cycle consisting of 5 s UV irradiation followed by a 30 s rest interval. (d) End‐to‐end manipulation of particles at the air‐water interface via photochemical Marangoni propulsion. e) Water channels with pump‐coated ends demonstrated sequential pumping and transportation of a single disk particle and (f) collective manipulation of microparticle clusters.

Having demonstrated that ONB‐HFIP/PVB motor composites can be processed into films and coatings, and that they undergo UV‐triggered photolysis to release HFIP as readily usable fuel in situ, we next implemented this responsive material system in a set of surface tension micropumps for flow generation and manipulation. These pumps consisted of ONB‐HFIP/PVB composite films that were either coated or attached to the boundary walls of liquid containers. Under UV illumination, chemical micropumps generated Marangoni flow due to the release of HFIP (thus decreasing surface tension of the surrounding liquid media on demand) (Figure 3a). To directly visualize this process, we coated one section of a petri dish wall with ONB‐HFIP/PVB and dispersed a thin layer of carbon black particles on the surface of deionized water. In rest conditions (UV light off), no flow or motion of particles was observed. Upon UV illumination, ONB‐HFIP molecules were photocleaved to release HFIP which diffused into the aqueous phase. Given that HFIP has a very low surface tension (γ = 14.7 mN m^−1^) and is highly effective at reducing water surface tension at low concentrations [22, 41], the released HFIP generated strong local surface tension gradients. These gradients produced Marangoni flows that cleared nearby carbon particles and provided distinct flow patterns, serving as a direct visualization of the chemical micropump. When the pump coating was illuminated with UV light, even short exposure (1–10 s) produced strong flows, with longer irradiation resulting in more pronounced patterns (Figure 3b, Figure S16 and Movie S1). In control experiments, a passive PVB film without ONB‐HFIP (i.e., polymer matrix without fuel carrier molecules) was coated at the opposite end of the petri dish. The control passive coating showed no pumping of generated flow patterns. These results confirmed that the observed pumping effect originates primarily from Marangoni flow activated by UV light and subsequent fuel photolysis (rather than non‐specific photothermal effects). Leveraging this active light‐based control, we demonstrated that the pump can be activated on demand and repeatedly operated over a number of on/off cycles (Figure 3c, Figure S17 and Movie S2). For example, using 5 s of UV irradiation followed by 30 s of rest intervals, the pump maintained consistent flow patterns over 60 cycles (Figure S18).

Building on the ability of the pump coatings to generate Marangoni flows, we next explored their capability to manipulate and transport objects on water surfaces. The active pump coating was applied to the ends of a CNC‐machined water channel that can be illuminated from either side (Figure 3d). Disc particles were placed on the water surface and, upon light‐activation of the pump on either side, the localized generation of HFIP produced regions of concentrated fuel and reduced surface tension adjacent to the pump on either side, yielding steep surface tension gradients between the illuminated and distal ends of the particle. This gradient generated a strong net driving force, propelling objects placed on the surface away from the illuminated end toward the opposite side until the concentration gradient dissipated. After a particle reached the far end of the channel, irradiation of the coating on the opposite side triggered a fresh release of HFIP, generating a reversed gradient that pushed the object back to its initial position (Figure 3e, Movie S3). The high volatility of HFIP ensured that it did not accumulate significantly at the interface, enabling sequential and repeated pumping operations. When both ends of the path were coated with motor composites, the two propulsion cycles were nearly identical in duration (t ≈ 15 s each), confirming minimal interference from residual HFIP between cycles. Also, control experiments with passive PVB coatings (without fuel carrier molecule) showed no motion of the particle, confirming the primary contributions from Marangoni flows and discarding significant contributions from photothermal effects (Figure S19). In addition to single‐object transport, we demonstrated collective manipulation of microparticle clusters using the same pumping mechanism. A cluster of glass beads with diameters of ∼500 µm was placed on the water surface within the channels. Upon irradiation, simultaneous HFIP release produced cooperative Marangoni flows, displacing the beads together as a unified cluster (Figure 3f, Movie S4). Control experiments with passive PVB coatings also showed no motion of particle clusters (Figure S20), confirming the active pumping from photochemical fuel release. This illustrates that the proposed photoresponsive chemical pump is not only capable of directional control for flow manipulation and directional transport of individual objects, but also scalable to ensemble manipulation of multiple particles, which can be integrated in active particle collectives and swarm microrobotic systems.

### 2.4 Photochemical Fuel for Aquatic Small‐Scale Robotics

**FIGURE 4:**
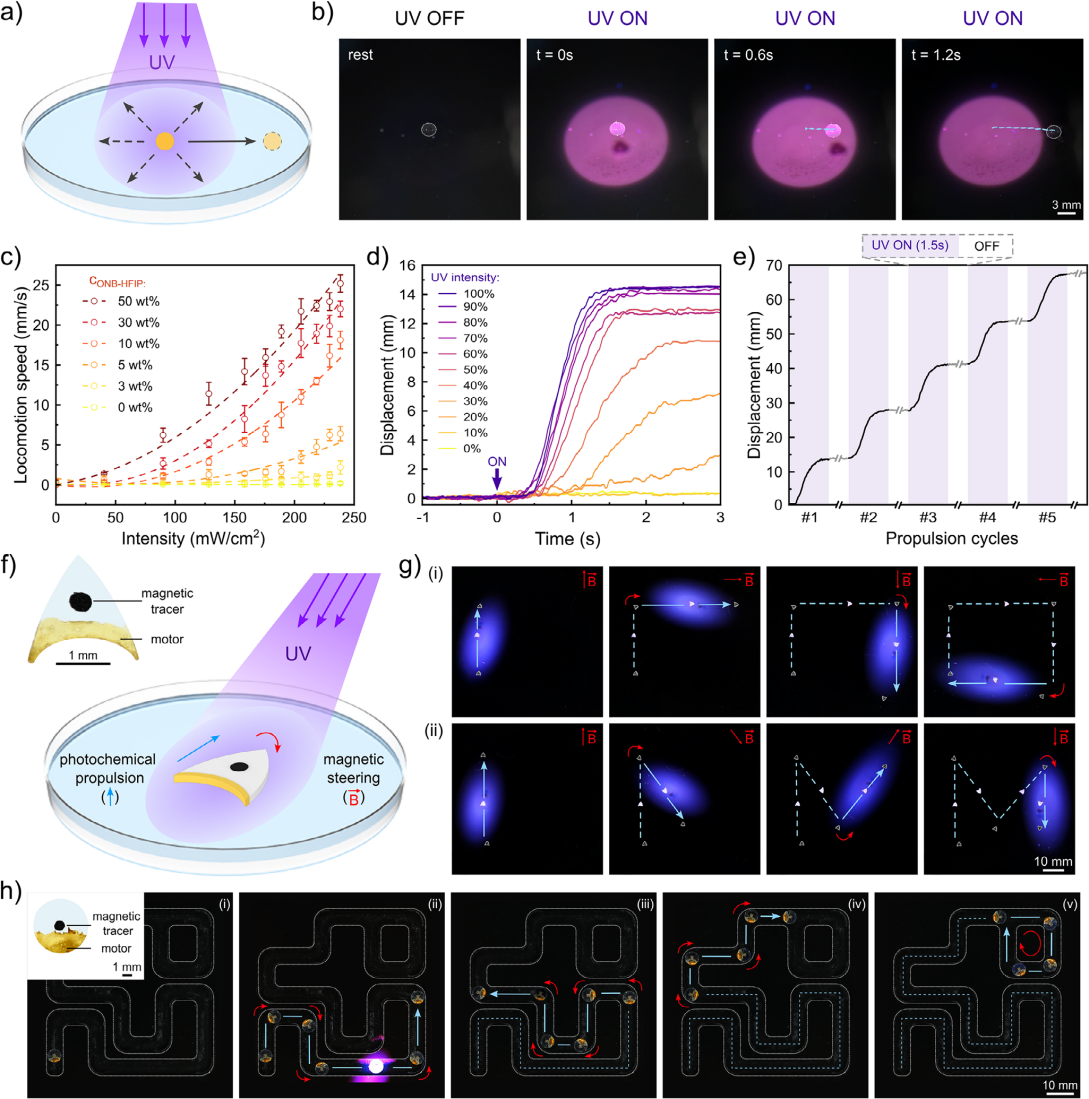
Photochemical propulsion for small‐scale swimming robots. (a) Schematic illustration and (b) optical images showing locomotion of ONB‐HFIP/PVB composite films propelled by UV‐triggered fuel release. (c) Motor particle performance evaluated by the locomotion speed of composite films with different mass loading of photolytic fuel‐carrier molecules. (d) Displacement of particles under different UV intensities with time, shown here for particles containing 30% (w/w) fuel‐carrier molecules. (e) Displacement of 30% (w/w) particles in cyclic on/off tests, with 1.5 s UV illumination within each cycle (1st–5th cycles shown here). (f) Design and mechanism of hybrid swimming robots, showing orthogonal combination of propulsion via photochemical fuel release and steering via magnetic control. (g) Locomotion trajectory control of hybrid swimming robots (l = 2 mm) following (i) rectangular and (ii) M‐shaped 2D paths. (h) Navigation of hybrid swimming robots (l = 5 mm) within a maze channel through sequential and repeated propulsion‐steering cycles.

In addition to pump coatings and films on static systems, ONB‐HFIP/PVB composites can also be processed into freestanding films and active particles where they act as a photochemical motor. Similar to the working mechanism of the pumps, free‐floating ONB‐HFIP/PVB films release HFIP fuel on demand upon UV illumination, generating Marangoni flows that self‐propel the particle itself (Figure 4a). A 3 mm diameter ONB‐HFIP/PVB particle was illuminated under a uniform UV spot (22 mm in diameter), which activated the particle self‐propulsion within 1 s of exposure and continued moving until leaving the illuminated region (Figure 4b, Movie S5). Control passive particles (lacking ONB‐HFIP molecules) showed no displacement under identical conditions, ruling out possible photothermal effects (Figure S21). To develop an optimal formulation of ONB‐HFIP/PVB motors, we studied photochemical particles with fuel carrier loadings of 0, 3, 5, 10, 30, and 50% (w/w) (Figure 4c, Figure S22). The particle velocity increased with concentration, with 50% (w/w) particles achieving speeds up to 25.2 mm s^−1^ at 237 mW cm^−2^. However, fuel utilization efficiency improved only marginally beyond 30% (w/w). Based on this trade‐off, 30% (w/w) motor particles were selected for subsequent experiments, as they balanced high propulsion performance with fuel efficiency. We further investigated the performance of these motor particles under varying UV intensities (Figure 4d). As UV irradiation intensity increased from 0 to 237 mW cm^−2^, locomotion speed rose correspondingly, reaching a maximum speed of 22 mm s^−1^. The motor particles also exhibited excellent durability, lifetime, and repeatable locomotion. After 50 on/off operation cycles, particles maintained locomotion speed up to ∼82% of their initial performance (Figure 4e, Figure S23). Prolonged illumination experiments at maximum intensity (237 mW cm^−2^) for 70 min still sustained high velocities without an apparent loss of performance (Figure S24). Additionally, ONB‐HFIP fuel carrier molecules processed into photochemical particles still retained their activity and locomotion speed after at least two years in “rest” ambient conditions (without UV illumination) (Figure S25). This high stability and on‐demand fuel release capabilities provides a wide operational window and lifetime as well as active control over speed and on/off modalities.

ONB‐HFIP/PVB can also be deposited as conformal motor coatings onto a broad variety of substrate materials, geometries, and length scales, providing facile surface integration with other microrobotic designs and actuation strategies. This approach simultaneously allows for endowing inactive objects with light‐responsive photochemical mobility as well as providing orthogonal control of robotic functions. To demonstrate this, we fabricated hybrid swimming robots from laser‐micromachined 150 µm polyethylene terephthalate (PET) films. These structures, with a characteristic length of 2 mm, were engineered with a low hydrodynamic drag profile and a concave posterior cavity to host the motor coating, inspired by previous work [22]. An ONB‐HFIP/PVB motor coating was applied to the posterior cavity to release the chemical fuel exclusively in this region and produce an anisotropic surface tension gradient that will propel the particle forward. Then, a magnetic tracer (polydimethylsiloxane/NdFeB composite thin film, magnetized anisotropically) was adhered on top of the structure to provide torque‐enabled rotational control via homogeneous magnetic fields (Figure 4f inset). The integration of these two actuation mechanisms provided real‐time decoupled and orthogonal control of the microrobot trajectory, combining photochemical propulsion (with on/off switching behavior) and magnetic steering (Figure 4f). Control experiments without carrier fuel molecules, the robot experienced only magnetic torque (rotation) but no translational motion, and that without magnetic tracers, the motor moves only through one direction regardless of the UV illumination direction, confirming the hybrid propulsion and steering mechanism and also ruling out possible artifacts from photothermal effects (Figure S26, Movie S6). Only through the orthogonal combination of propulsion and steering, we realized sequential propulsion and steering control (Figure S27). We have demonstrated this hybrid actuation and trajectory control approach in a custom electromagnetic coil system capable of generating variable magnetic fields in 360°. The hybrid robot was navigated along open‐loop programmable 2D paths, including square and M‐shaped trajectories (Figure 4g, Movie S7). To further highlight adaptability, we tested the hybrid actuation and control system in a confined maze channel filled with water. A 5 mm‐diameter disk robot was designed to homogenize rotational diameter and avoid jamming at corners. Similarly, a motor film was coated asymmetrically at the rear edge, enabling anisotropic forward propulsion under UV irradiation, and a magnetized tracer was used for steering control. At the corners, the robot was stopped by temporarily turning off UV illumination, and the external magnetic field was rotated to steer and turn the robot. Through repeated sequential propulsion–steering control cycles, the robot successfully navigated the entire maze path, demonstrating guided transport even in geometrically constrained environments (Figure 4h, Movie S8). This hybrid design and control strategy, combining photochemical propulsion and magnetic steering, provides a versatile platform for precise navigation of micromotors in complex environments.

## 3 Conclusions

The novel fuel carrier molecular design strategy reported here integrates two functional units into the same molecule: a photolabile *o*‐nitrobenzyl derivative (ONB) that triggers a bond cleavage reaction under controlled light stimuli, and an ultralow surface tension fragment (HFIP) that is released as fuel as a result of the photochemical bond scission process. The resulting ONB‐HFIP carrier system exhibits a photoresponsive behavior to release chemical fuel on demand and to locally manipulate surface tension generating Marangoni flows. This concept has three major advantages that enable their broad application to active matter and microrobotic materials design: (i) *Stable storage and on‐demand release*. The fuel unit as an individual component is volatile (evaporates easily) and is highly miscible with water, which leads to its rapid loss, leakage, and consumption. However, when incorporated in the carrier molecule, it is highly stable (solid form) and immiscible in water at room temperature while retaining its photochemical activity. Fuel carrier molecules were tested after stored at room temperature and ambient conditions for two years without a perceivable loss of activity or performance (Figure S25). This strategy (analogous to metabolic processes in biology) stores volatile fuel in a more stable form for long‐term storage, to be later consumed on demand for photochemical locomotive functions. (ii) *Processing and embodied energy*. The fuel carrier molecule can easily be dispersed in a polymer matrix to fabricate photoactive composites, and we have demonstrated its versatile processing via a variety of solution‐based methods such as film coating, casting, or molding. These processing methods can be used to fabricate complex structures and geometries on a variety of substrate materials. These composites can be applied with combined structural, energy storage, and motor functions, resulting in multifunctional and modular components that can be incorporated into a variety of small‐scale robots and devices. This approach is particularly useful in the miniaturization of robotic devices where size and weight are stringent design restrictions, as structural elements with distributed embodied energy can provide new solutions to resolve performance trade‐offs [17, 53]. (iii) *Marangoni flow control mechanisms*. Marangoni propulsion has been previously explored in multiple active matter and microrobotic systems, usually presenting passive trajectory control by: particle shape design [23, 54], active control by shape‐morphing [54, 55], and active control by steering with other actuation methods [5, 22]; and locomotion speed control by: passive diffusion control through material porosity (e.g., polymer hydrogels [23], metal‐organic frameworks [56], nanocrystalline materials [22], etc.) and active control by fuel delivery method (valves, syringes, pumps, microfluidics, etc.) [20, 21, 57]. These approaches, however, require impractical mechanisms and fuel reservoirs (which add volume and weight, reduce the autonomy and efficiency of the propulsion system, and hinder device miniaturization). The motor system proposed in this article presents a hybrid approach with active control of speed (with on/off control) via materials chemistry. Rather than requiring bulky external components, the presented approach relies on a photoresponsive molecular switch, therefore achieving on‐demand on/off behavior and regulatable speed by controlling the illumination conditions. Active speed control with on/off switching behavior also allows for an off “rest” state where fuel is not being consumed, which in turn prolongs the lifetime and efficiency of the system. Furthermore, the proposed motor system can be applied to a broad diversity of materials and structures across length scales, and therefore it is compatible with both passive and active trajectory control approaches. We demonstrated these properties in several devices that leverage the photoactive flow control of ONB‐HFIP fuel carriers, including Marangoni micropump systems for surface‐tension‐active particle transportation and untethered hybrid microrobots that combine photochemical on–off propulsion with magnetic steering for trajectory control. Due to its versatile processing and compatibility with other materials, these fuel carriers could be easily integrated into other microrobot architectures as modular structural fuel reservoirs and motors, thus opening new opportunities for embodied energy design in microrobotics. While the fuel carrier molecule described here has been developed for UV photolysis, the concept can be easily adapted to longer wavelength light (such as green or NIR) with different photoresponsive linkers, which could open applications in medicine by enhancing tissue penetration depth, efficiency, and overall biocompatibility.

## 4 Materials and Methods

### 4.1 Materials and Synthesis of ONB‐HFIP

4,5‐Dimethoxy‐2‐nitrobenzyl chloroformate, 1,1,1,3,3,3‐hexafluoro‐2‐propanol (HFIP), pyridine, polyvinyl butyral (PVB), dichloromethane (DCM), and tetrahydrofuran (THF) were purchased from Sigma–Aldrich. All regents and solvents were used as received without further purification.

To synthesize ONB‐HFIP, 1,1,1,3,3,3‐hexafluoro‐2‐propanol (0.18 g, 1.1 mmol, 1.5 equiv.) and pyridine (70 mg, 0.9 mmol, 1.2 equiv.) were added in sequence to a solution of 4,5‐dimethozy‐2‐nitrobenzyl chloroformate (200 mg, 0.7 mmol, 1.0 equiv.) in dry DCM (2 mL). After stirring for 16 h at 25°C, the reaction mixture was diluted with ethyl acetate (100 mL) and washed with water (3 × 100 mL). Then, the organic layer was combined, dried over sodium sulfate, filtered, and concentrated under reduced pressure. The resulting product was further purified by flash column chromatography (elution with 10% ethyl acetate in *n*‐hexane) to afford ONB‐HFIP as an orange solid (0.22 g, 0.53 mmol, 76%). ^1^H NMR (400 MHz, CDCl_3_): δ 7.78 (s, 1 H), 6.99 (s, 1 H), 5.75 (d, 2 H, *J* = 0.56 Hz), 5.64–5.59 (m, 1 H), 3.99–3.98 (d, 6 H, *J* = 5.9 Hz); ^13^C NMR (100 MHz, CDCl_3_): δ 153.89, 152.53, 148.68, 139.53, 125.09, 121.24, 118.99, 109.23, 108.31, 70.47, 68.51, 56.41, 56.37; IR (cm^−1^): 1780, 1525, 1326, 1247, 1219, 1192; HRMS (TOF MS FD+, *m/z*): calcd. for C_13_H_11_O_7_F_6_ (M^+^) 407.04342; found: 407.04344.

To fabricate ONB‐HFIP/PVB composites, PVB powder was dissolved in THF at a concentration of 20 mg ml^−1^. The PVB solution was then mixed with ONB‐HFIP powders at specific mass concentrations (0%, 3%, 5%, 10%, 30%, and 50%) and thoroughly mixed to form a motor composite solution. Then, 100 µL of PVB solution or motor composite solution were cast on Teflon substrates and left to evaporate in the fume hood, producing even films with thickness of 0.2 mm with different concentrations.

### 4.2 Structural Characterization

High‐performance liquid chromatography (HPLC) spectra were obtained using an analytical reversed‐phase HPLC (Hitachi 5000 series Chromaster). The column was a TSKgel ODS‐100Z C18 column (7.5 cm × 4.6 mm, 5 µm particle size). The column was equilibrated with 8:2 CH_3_CN–H_2_O mixture as a mobile phase at a flow rate of 1 mL min^−1^. After each analysis, the column was thoroughly washed with CH_3_OH for 10 min. UV‐vis spectra were observed by an Optizen 2120 UV spectrophotometer with a quartz cuvette with a 10‐mm path length. Proton nuclear magnetic resonance (^1^H NMR) and carbon nuclear magnetic resonance (^13^C NMR) spectra were recorded using a Bruker Ascend 400 MHz NMR spectrometer at 25°C, processed with a MestReNova software. Proton chemical shifts are expressed in parts per million (ppm, δ scale) and are referenced to tetramethylsilane ((CH_3_)_4_Si 0.00 ppm) or to residual protium in the NMR solvent (CDCl_3_, δ 7.26 ppm). Data are represented as follows: chemical shift, multiplicity (s = singlet, d = doublet, m = multiplet and/or multiple resonances), integration, and coupling constant (*J*) in hertz. Carbon chemical shifts are expressed in parts per million (ppm, δ scale) and are referenced to the carbon resonance of the NMR solvent (CDCl_3_, 77.23 ppm). The structure of the motor composite was analyzed by Fourier Transform Infrared (FTIR) spectroscopy in a ThermoFisher Nicolet iS20 spectrometer with an Attenuated Total Reflection (ATR) accessory at 128 scans and 4 cm^−1^ resolution per spectrum. The resulting FTIR spectra were baseline‐corrected and normalized to the O‐H band. The peaks from these tests were tracked as a function of irradiation time and were analyzed using a linearized exponential decay function with the following equation:

ln[A]=ln[A0]−kt

where ln[A] is the natural logarithm of the normalized peak areas or intensities at time t, ln[A_0_] is the corresponding initial value, and *k* is the apparent pseudo‐first‐order rate constant. Linear regression was performed using OriginPro, and *k* was obtained from the negative slope of the fitted curve.

### 4.3 Mechanical Characterization

Abrasion tests were conducted using a Taber Linear abrader (5750, Taber manufacturer) according to ASTM standard D1044. A CS‐5 abrasive tip was used to create scratches over the PVB film surface under normal loads of 3N over 0.5‐inch distance at a constant frequency of 60 cycles min^−1^. Stress‐stain curves were tested using a texture analyzer (TA.HD Plus C, Stable Micro Systems). The PVB films or ONB‐HFIP/PVB composite films were cut using a dog bone die cutter following the design of ASTM standard D1708 and then tested with the distance between the upper and lower grips as 10 mm and the strain rate of 60 mm min^−1^.

### 4.4 Pumping Tests

A 20 µL motor composite solution (30% w/w) and PVB solution were cast on the opposite ends of a petri dish and left to dry overnight in the fume hood, which was repeated 5 times to achieve a thick point‐coating. Then the petri dish was filled with DI water, followed by spreading a very thin layer of carbon black powder (Sinopia) on the water surface. An OmniCure S2000‐Elite UV Curing System with a UV filter (320–390 nm) and 5 mm light guide (P/N 805‐00002) was used for pumping tests. All pumping and locomotion videos were recorded with a Nikon Z6 III camera.

In pumping experiments in channels, a Bantam Tools desktop CNC milling machine with a 1/8″ flat end mill was used to machine all channels from poly (methyl methacrylate) (PMMA) sheets (9 mm thick) following the designed manufacturing process by Fusion, producing a channel width as 6 mm and length as 60 mm. To encapsulate channel ends, 1 mm thick glass slides (Fisherbrand) with either motor composite (30% w/w) or PVB point‐coatings were stuck to channels end with hot glue. When left to dry, channels will be filled with DI water for pumping tests. OmniCure was used with UV filter and light guide to achieve point‐concentrated UV illumination. Round, passive particles (diameter of 5 mm) were fabricated from PET films by laser machining. A 25 W Universal Laser Systems CO_2_ laser cutter (5% power, 10% speed, 1000 ppi, 1.5″ optics) was used to cut all passive particles following the specified CAD model and length scale from polyethylene terephthalate (PET) films (0.15 mm thick). Particle clusters were composed of glass beads (diameter of 500 µm).

### 4.5 Motor Propulsion Performance Characterization

Round disk‐shaped motor particles were fabricated by cutting motor composite films using an EMS‐Core sampling tool with a diameter of 3 mm. They were then transferred to petri dishes with DI water. Above the water surface, OminCure with a collimator was mounted to achieve a round and even light spot with a diameter of 22 mm. When the working distance is 60 cm, the light intensity of the light spot is adjustable from 0 to 237 mW cm^−2^. All locomotion trajectories and speed were analyzed with Tracker software.

Magnetoactive particles were fabricated from PET films by laser machine into bullet shapes (length scale as 2 mm) and disk shapes (diameter as 5 mm). Then the motor composite solution was applied to the posterior end of passive particles and left to dry overnight. Ferromagnetic microparticles (NdFeB, Magnequench MQFP‐15‐7‐D50) were mixed with PDMS in a 1:1 (w/w) ratio and cast into 200 µm thick films. The films were cut with EMS‐Core sampling tool, with diameters of 0.5 mm and 2 mm for bullet‐shaped particles and dish‐shaped particles, respectively. With magnetic film stuck to the center, coated particles were magnetized in a 1 T external magnetic field generated from VSM in a direction parallel to sample symmetric axis from coated end to non‐coated end. A pair of electromagnetic coils was mounted on two ends of a 3D‐printed rotating stage, leaving a free space in the center for a stationary stage. Then, a free‐rotating magnetic field (5 mT) was generated around the static stage, where the petri dish and channels with DI water were placed. Motor‐coated, magnetic‐active particles were transferred to the water surface, propelled by UV illumination from OmniCure and steered by magnetic field.

## Author Contributions

H.K. and A.P.F. conceived the idea, designed the study, and provided overall supervision. S.J. and J.W.K. synthesized and characterized the ONB‐HFIP molecule. C.H. fabricated and characterized the ONB‐HFIP/PVB composites and developed and characterized the pumping system, motor system, and hybrid swimming robots. K.M. assisted in the mechanical characterization of PVB films. Z.Z. assisted in developing the magnetic coil system for robot control experiments. All authors participated in manuscript revisions, discussions, and interpretation of the data.

## Conflicts of Interest

The authors declare no conflicts of interest.

## Supporting information




**Supporting File 1**: adma72499‐sup‐0001‐SuppMat.pdf.

**Supporting File 2**: adma72499‐sup‐0002‐MovieS1.mp4.

**Supporting File 3**: adma72499‐sup‐0003‐MovieS2.mp4.

**Supporting File 4**: adma72499‐sup‐0004‐MovieS3.mp4.

**Supporting File 5**: adma72499‐sup‐0005‐MovieS4.mp4.

**Supporting File 6**: adma72499‐sup‐0006‐MovieS5.mp4.

**Supporting File 7**: adma72499‐sup‐0007‐MovieS6.mp4.

**Supporting File 8**: adma72499‐sup‐0008‐MovieS7.mp4.

**Supporting File 9**: adma72499‐sup‐0009‐MovieS8.mp4.

## Data Availability

The data that support the findings of this study are available in the supplementary material of this article.
