# Supplementary material for: Photochemical Fuel Carrier Molecules for Robotic Embodied Energy
Source: Adv Mater. 2026 Feb 28;38(23):e20447. doi: 10.1002/adma.202520447 (PMC13103627; doi:10.1002/adma.202520447)
Supplement: Supplementary file 1 — Supporting File 1: adma72499‐sup‐0001‐SuppMat.pdf. [file ADMA-38-e20447-s006.pdf]

Supplementary Information

Photochemical Fuel Carrier Molecules for Robotic Embodied Energy

Chuqi Huang[1,†], Songah Jeong[2,†], Ji Woo Kim[2], Kanyarat Mantala[1], Zenghao Zhang[1], Hyungwoo Kim[2,*], Abdon Pena-Francesch[1,3,4,5,6,*]

[1]Department of Materials Science and Engineering, [3]Department of Chemical Engineering, [4]Macromolecular Science and Engineering, [5]Robotics Institute, [6]Biointerfaces Institute, University of Michigan, Ann Arbor, MI 48109, USA

[2]School of Polymer Science and Engineering, Chonnam National University, 77 Yongbong-ro, Buk-gu, Gwangju, 61186, South Korea

[†] C.H. and S. J. contributed equally to this work.

*Corresponding: *kimhw@jnu.ac.kr*, *abdon@umich.edu*

Contents in this SI file:

Figure S1 | ^{1}H NMR spectrum of ONB-HFIP

Figure S2 | ^{13}C NMR spectrum of ONB-HFIP

Figure S3 | FTIR spectrum of ONB-HFIP

Figure S4 | Color change of ONB-HFIP in solutions

Figure S5 | Photolytic reaction conversion under incandescent light

Figure S6 | Solid-state photocleavage of ONB-HFIP powder

Figure S7 | Transparent polymer matrices for ONB-HFIP encapsulation

Figure S8 | PVB polymer films

Figure S9 | Stress-strain curves of ONB-HFIP/PVB composite films

Figure S10 | Infrared spectrum of ONB-HFIP/PVB composites

Figure S11 | Byproduct stability

Figure S12 | Time-resolved kinetic study of photocleavage and fuel release

Figure S13 | Photolytic kinetics under varied UV intensities

Figure S14 | Experimental NMR procedure for solid-state photocleavage

Figure S15 | ^{1}H NMR spectrum of ONB-HFIP/PVB composites

Figure S16 | Flow patterns during pumping tests

Figure S17 | 1st 2 cycles of on/off pumping tests

Figure S18 | Cyclic pumping tests

Figure S19 | Single particle pumping

Figure S20 | Particle cluster pumping

Figure S21 | Particle locomotion under localized UV illumination

Figure S22 | Time-dependent displacement of particles

Figure S23 | Cyclic motor tests

Figure S24 | Sequential illumination tests on both sides of a composite film

Figure S25 | Storage stability

Figure S26 | Control experiments for hybrid swimming robots

Figure S27 | Decoupled propulsion and steering of a hybrid swimming robot

Other supplementary materials for this manuscript:

Movie S1 | Flow patterns during pumping tests

Movie S2 | Cyclic pumping tests

Movie S3 | Single particle pumping

Movie S4 | Particle cluster pumping

Movie S5 | Particle locomotion under localized UV illumination

Movie S6 | Hybrid swimming robots (control experiments)

Movie S7 | Locomotion trajectory control of hybrid swimming robots

Movie S8 | Navigation of a hydride swimming robot in confined environments

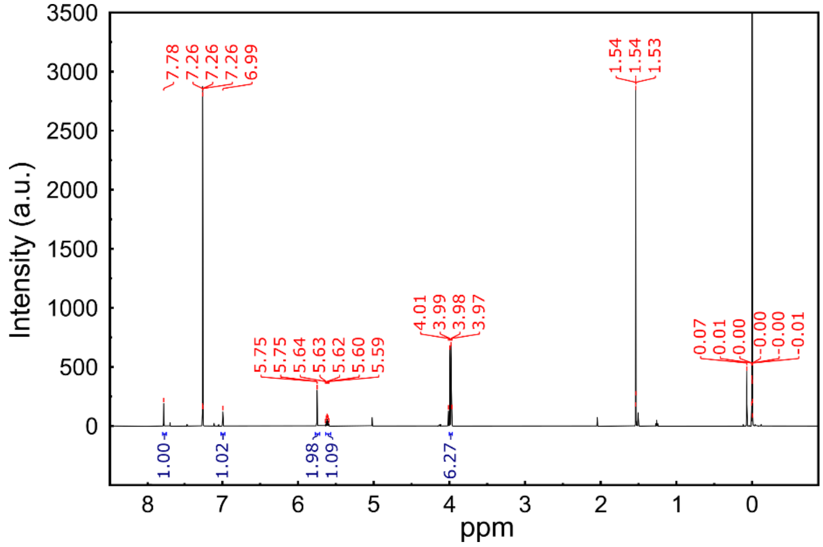

Figure S1 | ^{1}H NMR spectrum of ONB-HFIP.

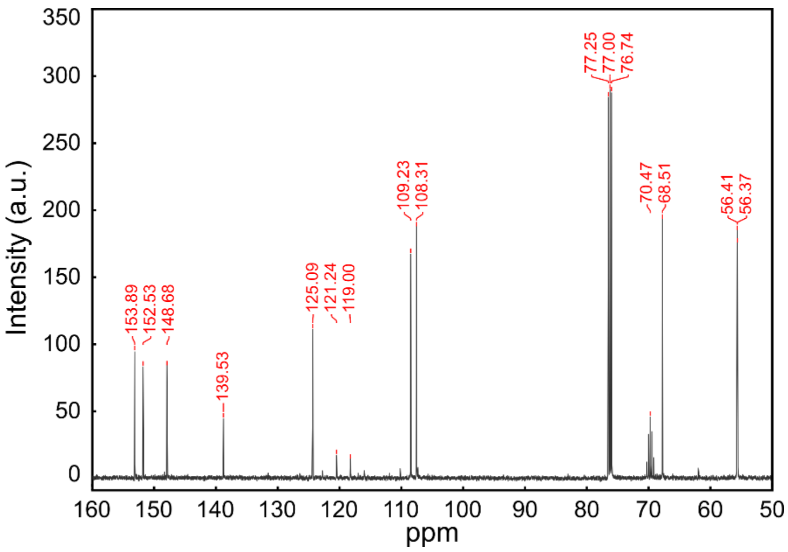

Figure S2 | ^{13}C NMR spectrum of ONB-HFIP.

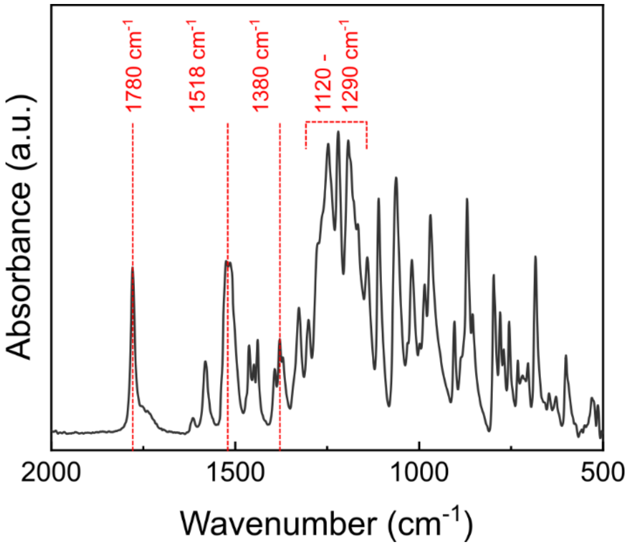

Figure S3 | FTIR spectrum of ONB-HFIP.

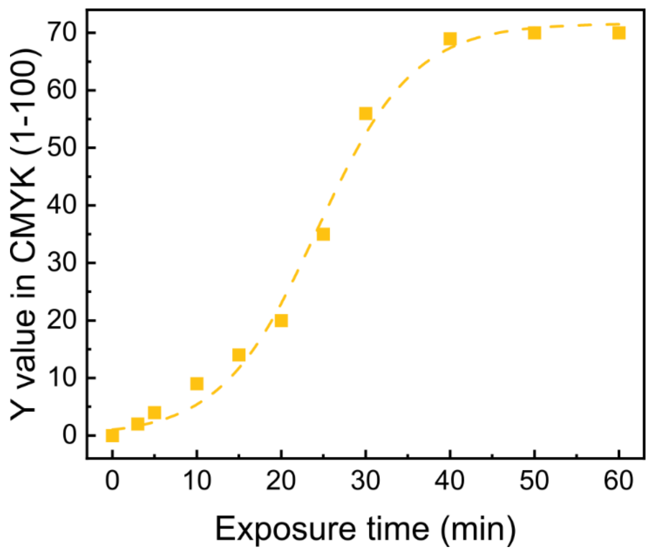

Figure S4 | Color change of ONB-HFIP in solutions as a function of varying UV exposure time, represented using Y value in the CMYK color model.

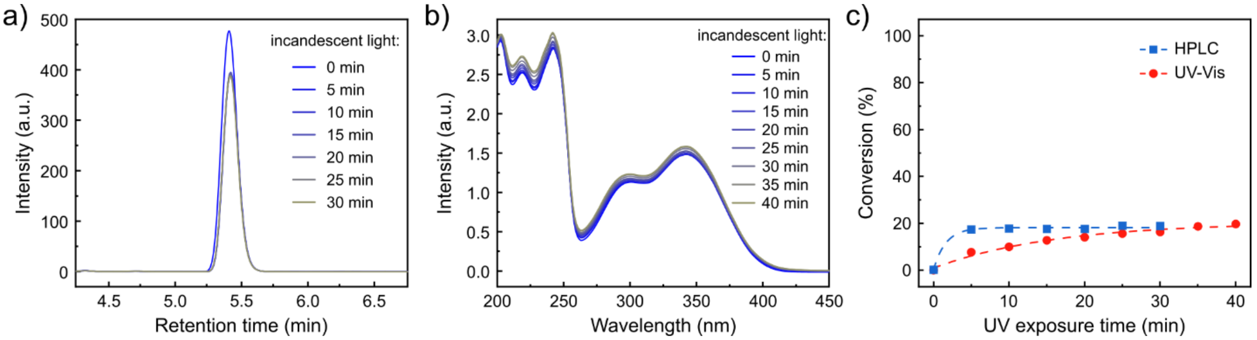

Figure S5 | Photolytic reaction conversion under incandescent light. a-b) Time-dependent changes in **a)** HPLC chromatogram and **b)** UV–Vis absorption spectra under incandescent light irradiation. **c)** Conversion degree of photolytic reaction, estimated from the changing ratio of HPLC peak areas and UV–vis absorption intensities at 343 and 264 nm over time under incandescent light irradiation.

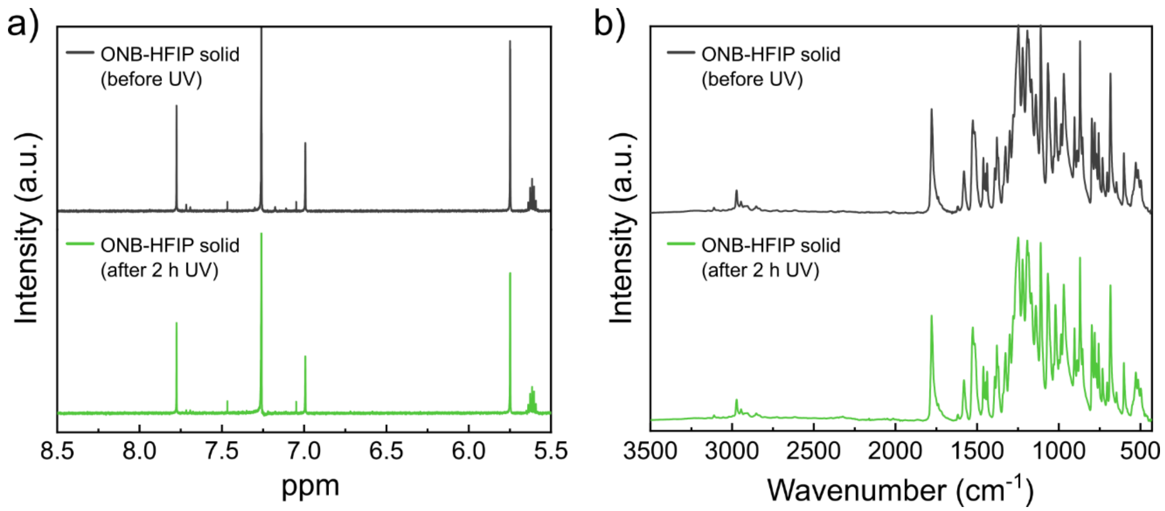

Figure S6 | Solid-state photocleavage of ONB-HFIP powder. a) Infrared spectrum and **b)** NMR spectrum of solid ONB-HFIP powder before and after 2 h of UV illumination, showing negligible changes.

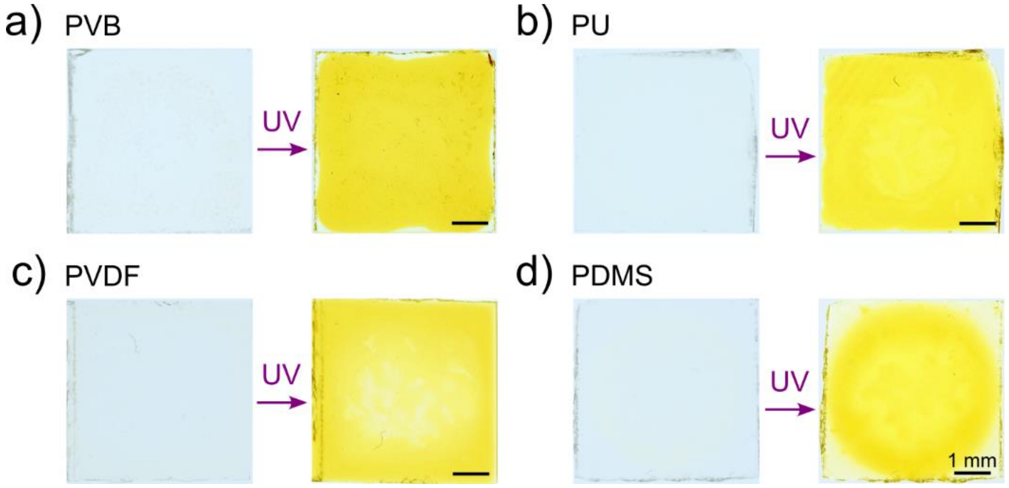

Figure S7 | Transparent polymer matrices for ONB-HFIP encapsulation, including **a)** polyvinyl butyral (PVB), **b)** polyurethane (PU), **c)** polyvinylidene fluoride (PVDF), and **d)** polydimethylsiloxane (PDMS).

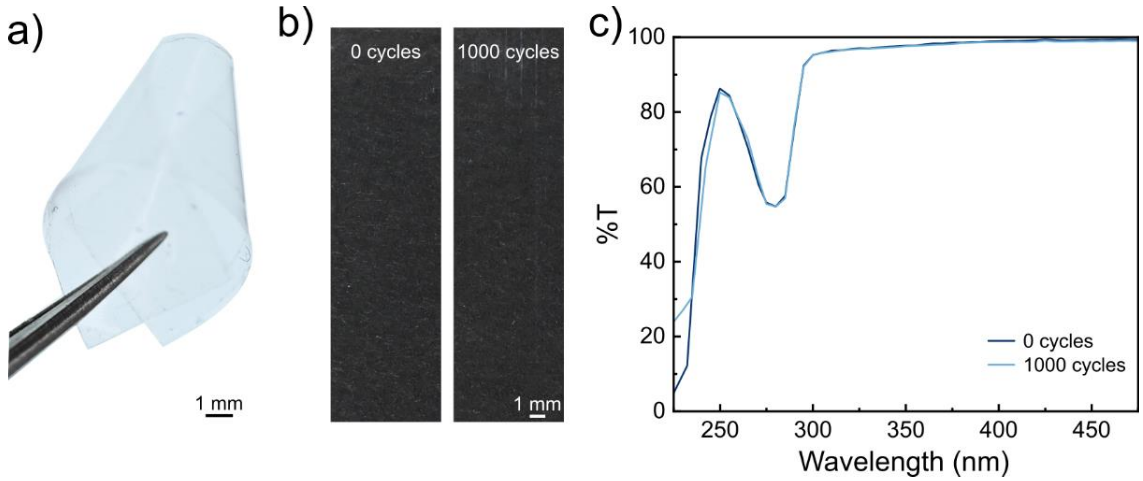

Figure S8 | PVB polymer films. a) PVB cast films are flexible in free-standing form. **b)** PVB films before and after 1000 Taber abrasion cycles. **c)** UV-vis spectra of PVB films before and after 1000 abrasion cycles, showing nearly 100% transmittance in the actuation wavelength range (320 - 390 nm).

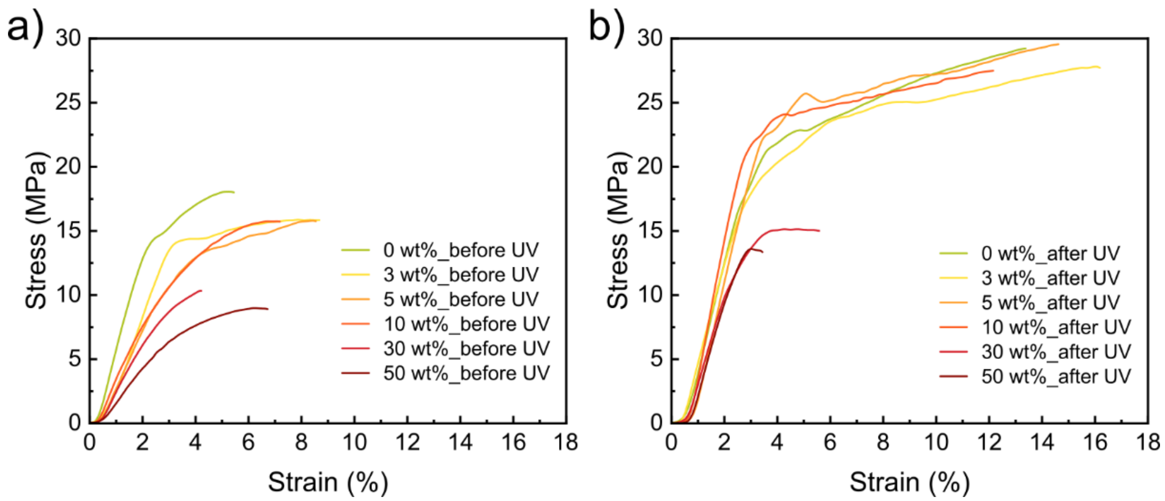

Figure S9 | Stress-strain curves of ONB-HFIP/PVB composite films with different mass loadings (0, 3, 5, 10, 30, 50 w/w%) **a)** before and **b)** after 70-min UV irradiation.

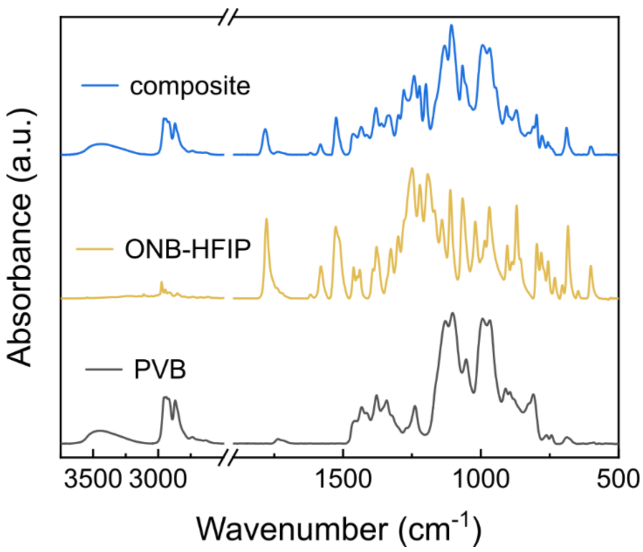

Figure S10 | Infrared spectrum of ONB-HFIP/PVB composites reveals successful integration and effective dispersion of ONB-HFIP molecules within PVB polymer matrix through the presence of characteristic absorption peaks from both ONB-HFIP (1782 cm^{-1} from C=O stretching in the carbonate group, 1525 cm^{-1} and 1336 cm^{-1} from asymmetric and symmetric N-O stretching in the nitro group, 1105 cm^{-1} and 1195 cm^{-1} for C-F stretching and 688 cm^{-1} for C-F symmetric deformation in trifluoromethyl group) and PVB (broad band at 3425 cm^{-1} for O-H stretching, 2700-2200 cm^{-1} for CH3, CH2, and CH stretching, and 1740 cm^{-1} for C=O stretching in ester group).

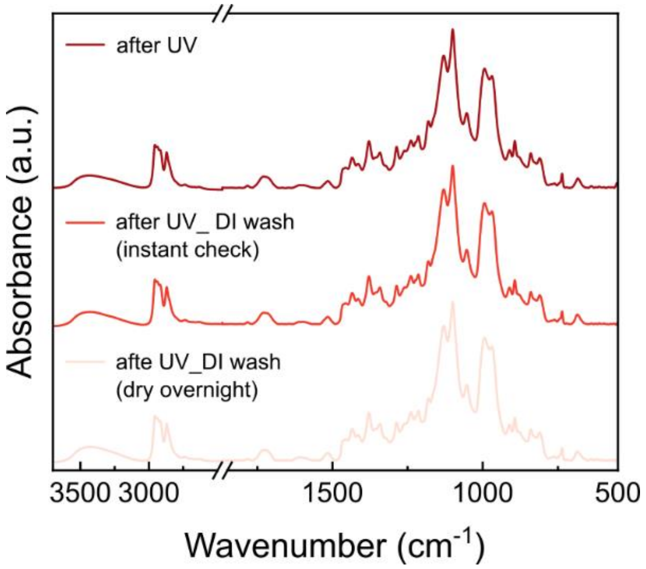

Figure S11 | Byproduct stability, proven through FTIR spectra of a composite film (top) immediately after UV irradiation, and after 30-min DI immersion (middle) followed by immediate FTIR measurement, and (bottom) followed by drying at room temperature overnight.

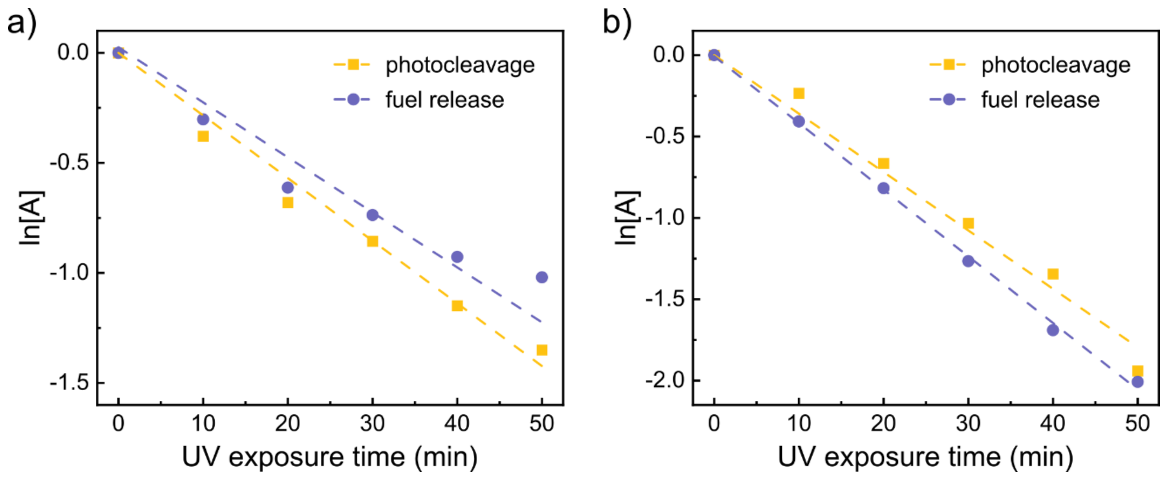

Figure S12 | Time-resolved kinetic study of photocleavage and fuel release, analyzed from peak area changes in **a)** infrared spectrum and **b)** NMR as a function of UV exposure time. Data were fitted to a pseudo-first-order kinetic model using a linearized exponential decay function: $\ln[A] = \ln[A_0] - kt$. Apparent rate constants were estimated from the slope of the fitted curve, with $k \approx 0.03$ min^{-1} with 95% *CI*: 0.0275-0.0325 min^{-1} (infrared) and $k \approx 0.04$ min^{-1} with 95% *CI*: 0.0371-0.0429 min^{-1} (NMR).

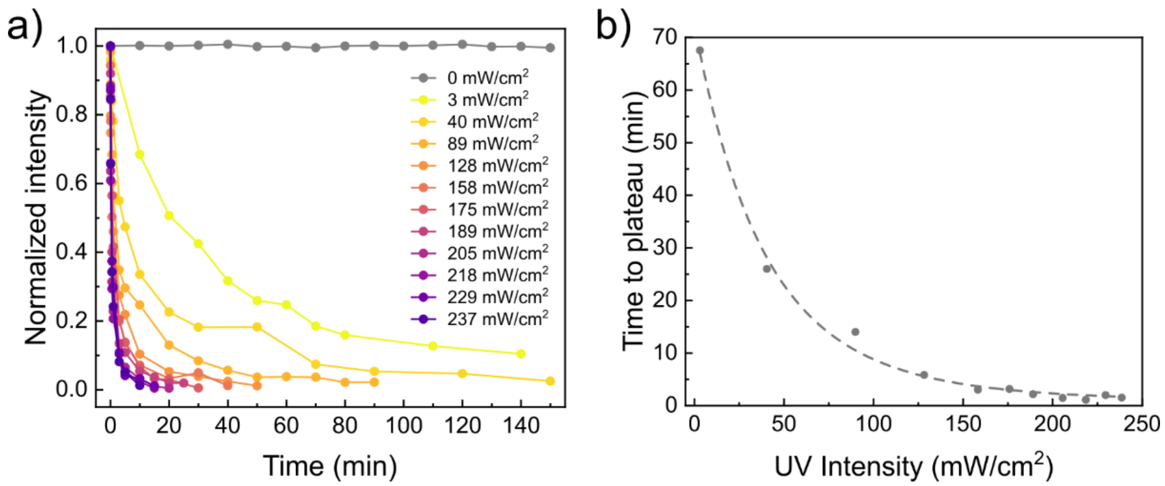

Figure S13 | Photolytic kinetics under varied UV intensities. **a)** Time-resolved changes in photocleavage under different UV intensities, estimated by changes in FTIR peak areas of C=O stretching in the carbonate group, **b)** Time to plateau state (as 80% photocleavage) as a function of UV intensity.

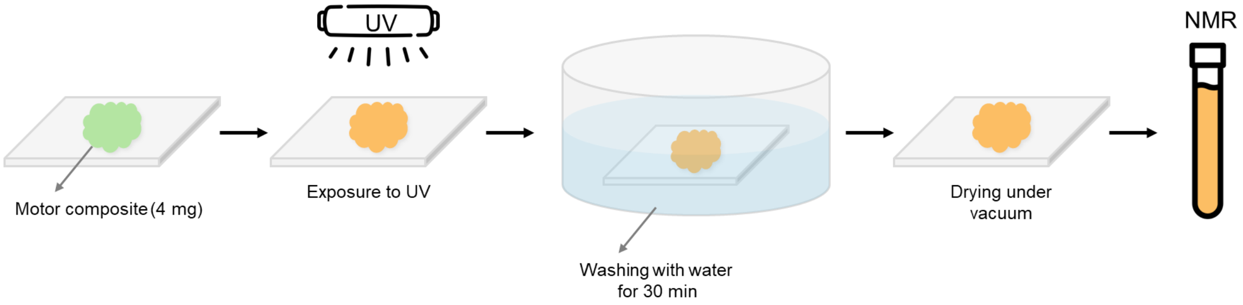

Figure S14 | Experimental NMR procedure for solid-state photocleavage of ONB-HFIP/PVB composites.

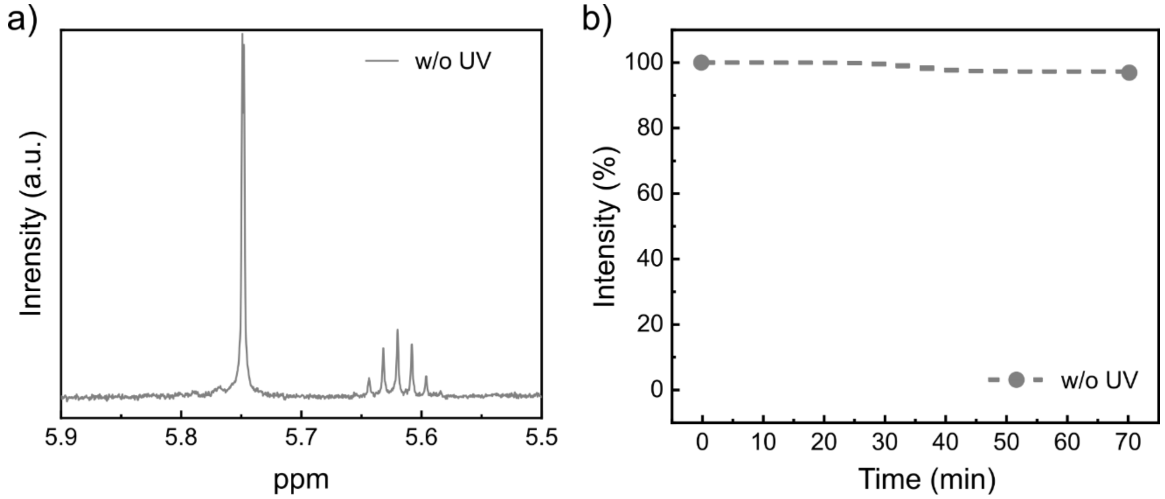

Figure S15 | ^{1}H NMR spectrum of ONB-HFIP/PVB composites. a) The magnified NMR spectra, highlighting peak *a* (the benzylic protons in ONB-HFIP at 5.75 ppm) and peak *b* (the tertiary hydrogen of HFIP at 5.62 ppm) of ONB-HFIP/PVB composites. **b)** Changes in NMR peak areas of *a* and *b* peaks as a function of immersion time in water, without UV irradiation.

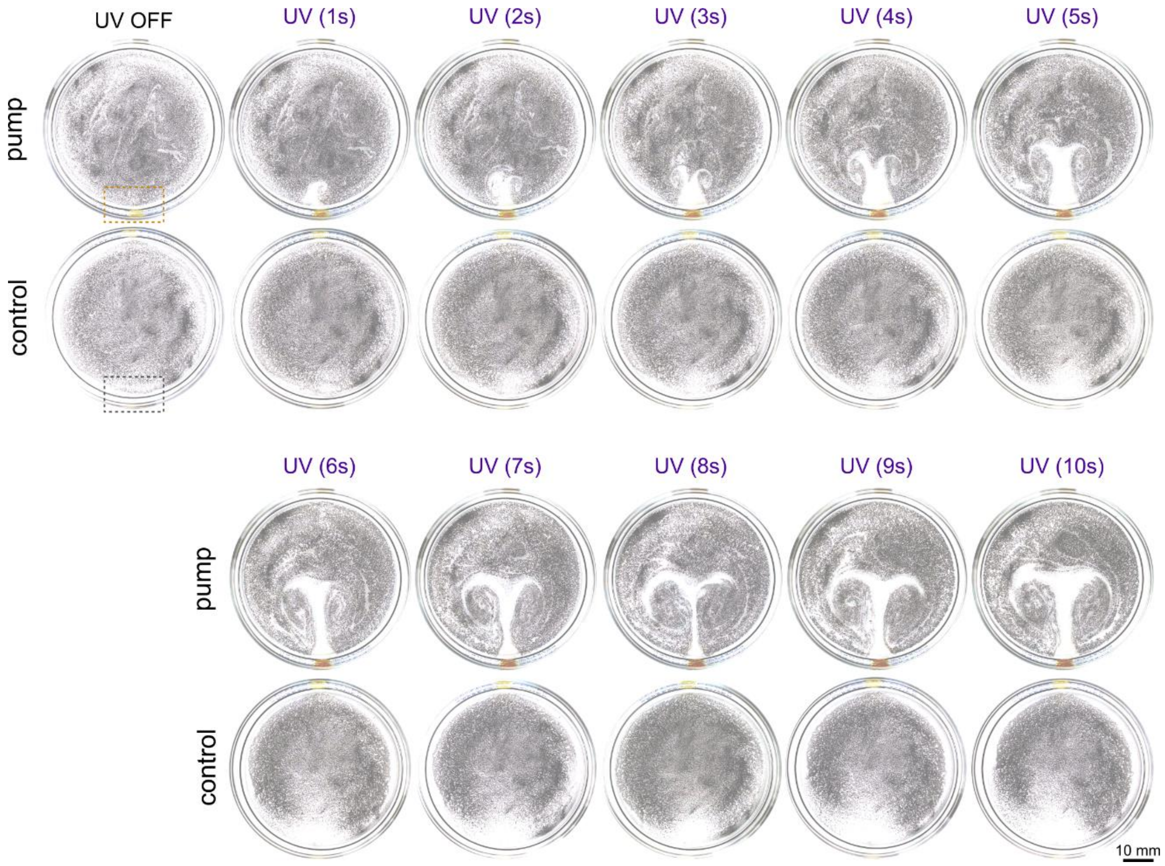

Figure S16 | Flow patterns during pumping tests, shown before UV irradiation and after different UV illumination durations (1-10s).

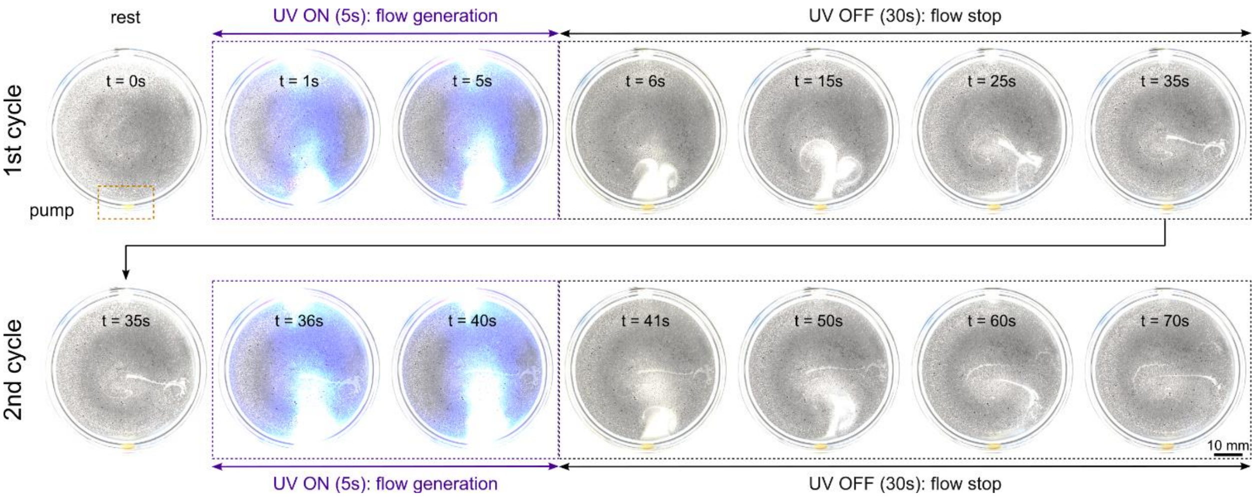

Figure S17 | 1st 2 cycles of on/off pumping tests, with each cycle consisting of 5 s UV irradiation to induce flow, followed by a 30 s interval allowing the system to return to rest.

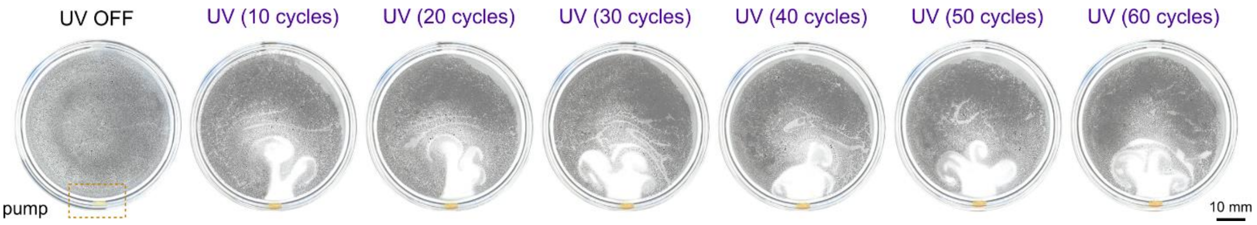

Figure S18 | Cyclic pumping tests. Flow patterns recorded before UV irradiation and after repeated illumination cycles (5 s UV on, 30 s off). The consistent flow patterns observed over 60 cycles demonstrate the repeatability and robustness of the photochemical micropump system.

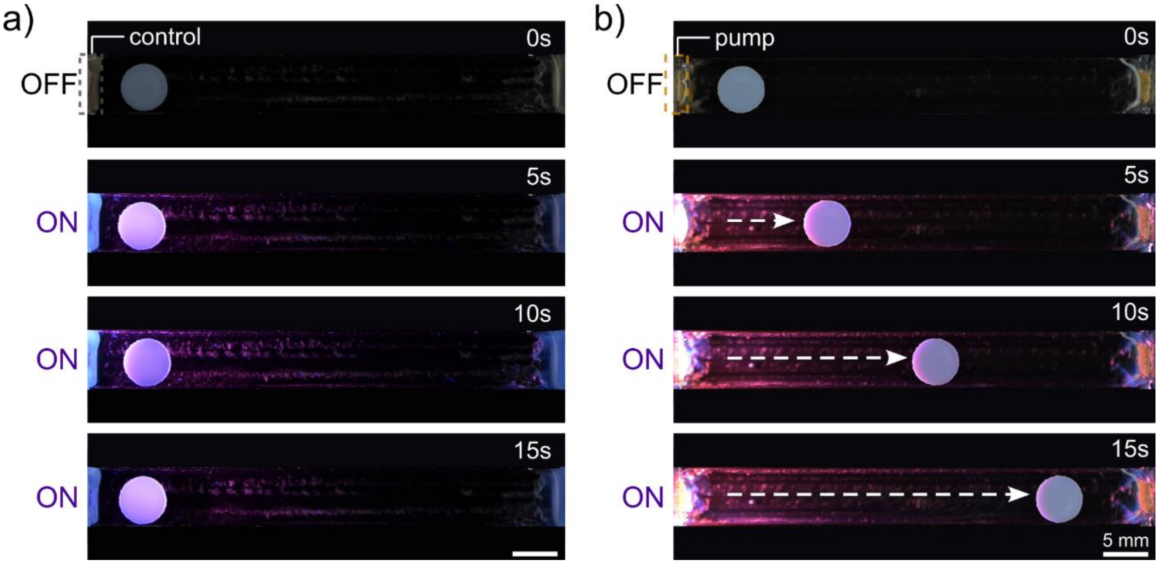

Figure S19 | Single particle pumping. Particles in a channel with **a)** passive PVB-coated ends showed no locomotion, whereas in a channel with **b)** active pump-coated ends demonstrated locomotion though the channel.

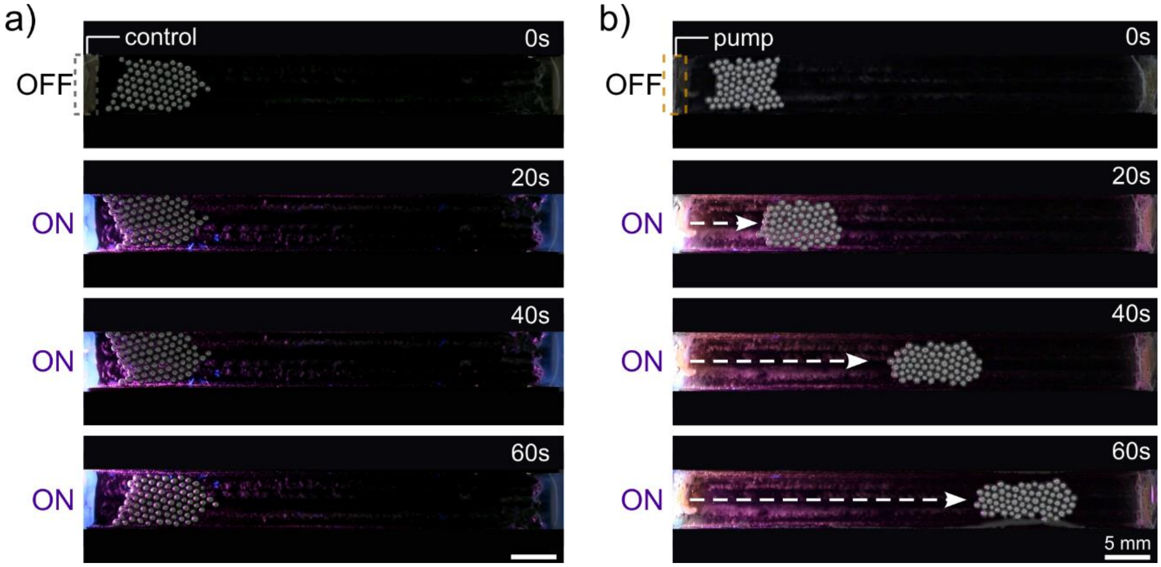

Figure S20 | Particle cluster pumping. Particle clusters in a channel with **a)** passive PVB-coated ends showed no locomotion, whereas in a channel with **b)** active pump-coated ends demonstrated locomotion though the channel.

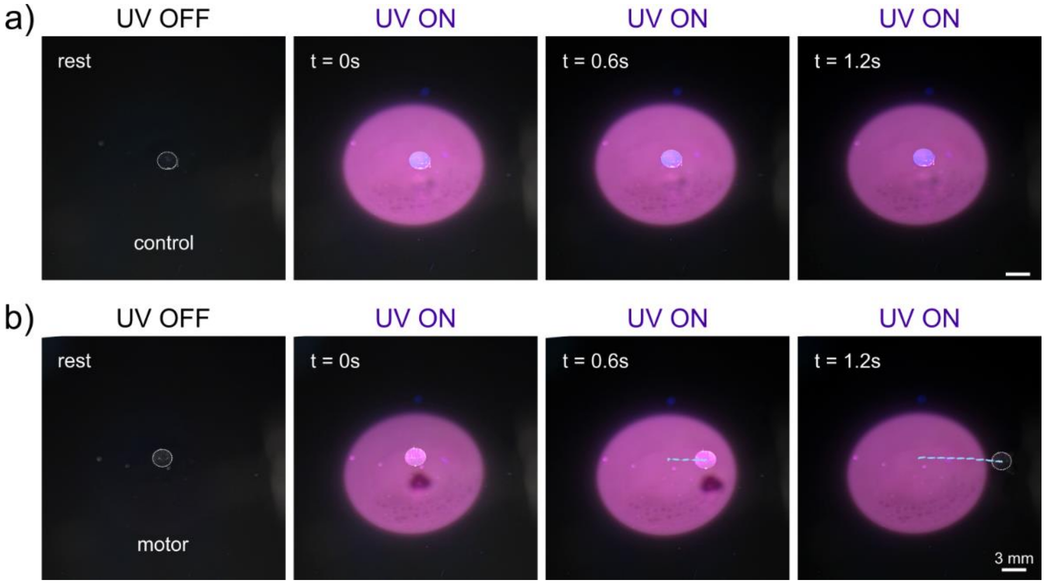

Figure S21 | Particle locomotion under localized UV illumination. a) A control particle from PVB film showed no locomotion, whereas **b)** a motor particle from ONB-HFIP/PVB composite film moved from the UV illuminated region to the unilluminated region, propelled by UV-triggered fuel release.

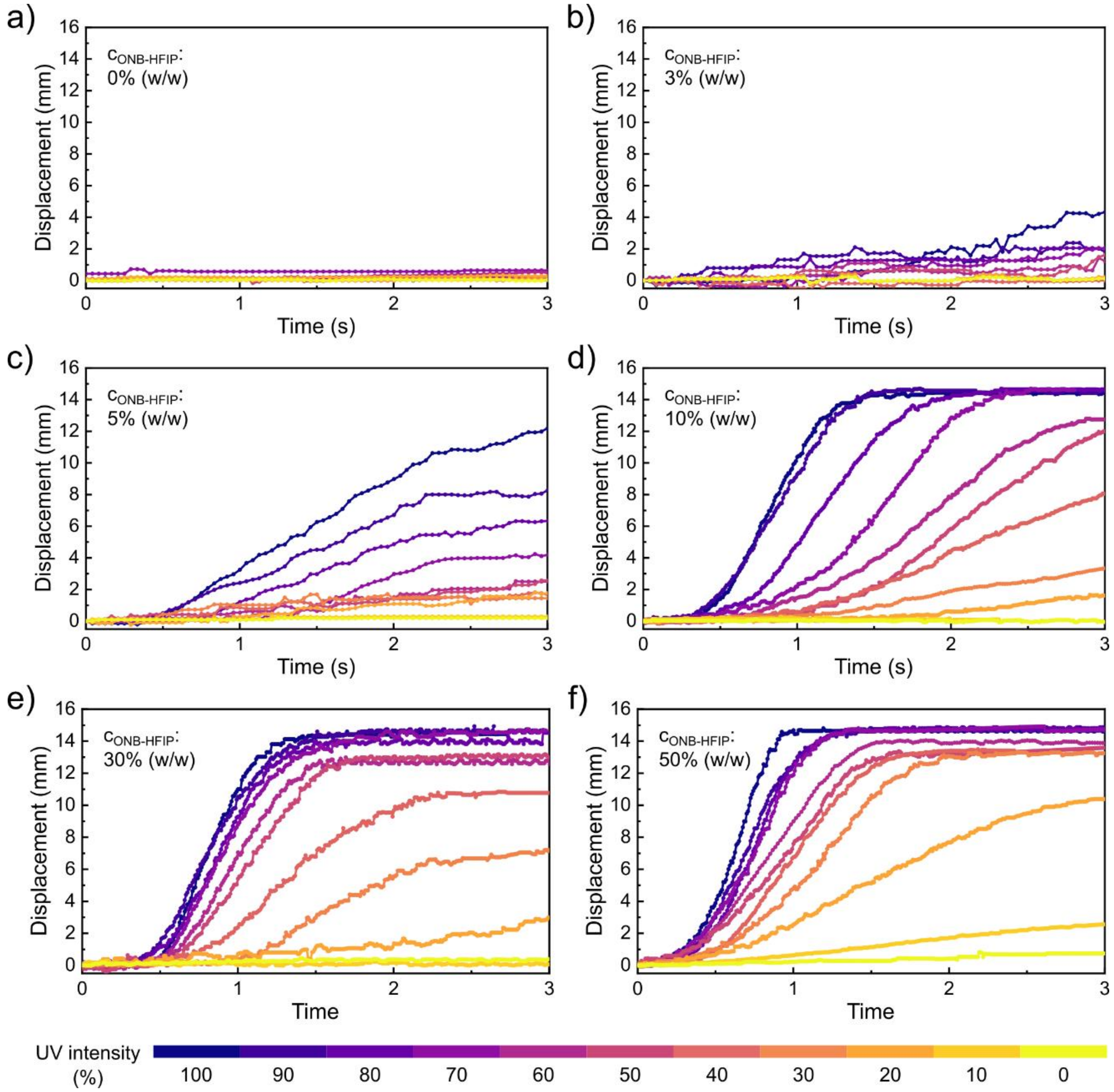

Figure S22 | Time-dependent displacement of particles under varying UV intensities, and with increasing mass loadings of ONB-HFIP: **a)** 0%, **b)** 3%, **c)** 5%, **d)** 10%, **e)** 30%, **f)** 50% (w/w).

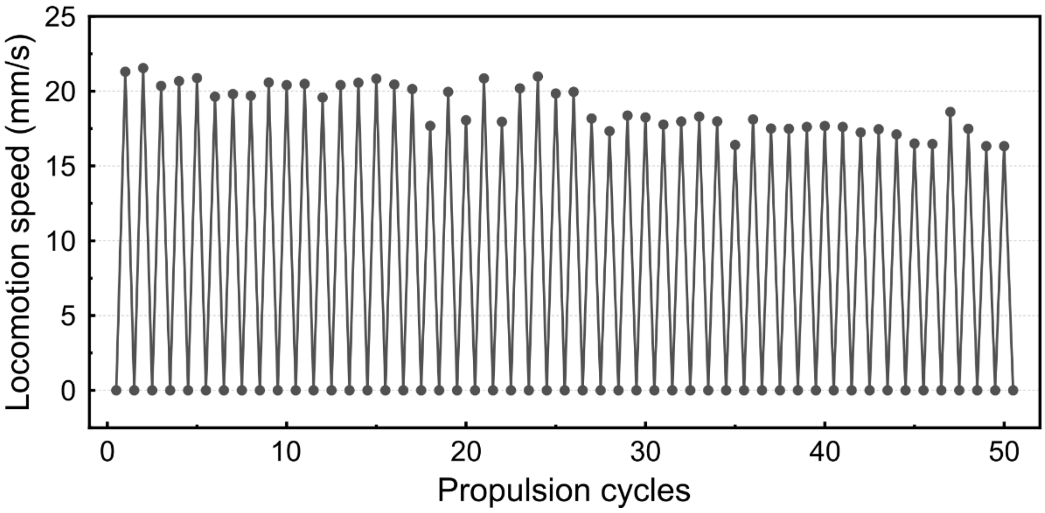

Figure S23 | Cyclic motor tests. Locomotion speed of particles containing 30% (w/w) ONB-HFIP was recorded over 50 consecutive on/off cycles, with 1.5 s UV irradiation applied in each cycle, demonstrating stable and repeatable photoactivated propulsion.

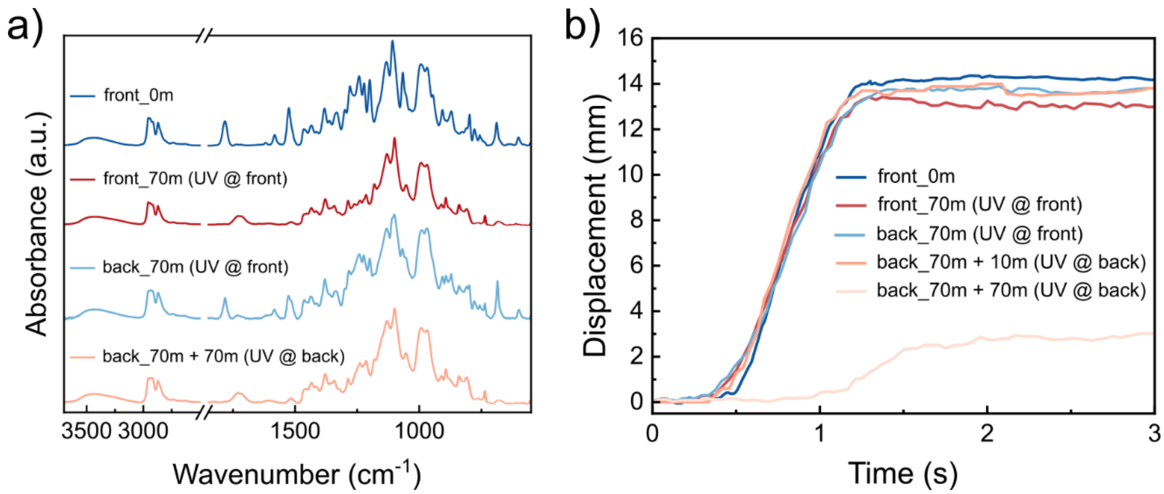

Figure S24 | Sequential illumination tests on both sides of a composite film. a) FTIR spectra and **b)** displacement as a function of time under UV of an ONB-HFIP/PVB composite film on both the front and back surfaces after sequential UV illumination. Front_0m presents a fresh particle, showing characteristic peaks of unreacted ONB-HFIP fuel carrier molecules and a locomotion speed as 22 mm s^{-1}. After 70-min UV illumination on the front side (front_70m UV @ front), FTIR spectra indicates photocleavage and HFIP depletion and minimal speed changes. At the 70-min point, however, back surface (back_70m UV @ front) presents negligible changes in both FTIR and locomotion, indicating limited photolysis on the back surface and a high concentration of unreacted molecules remaining through the film thickness. Only after extensive UV illumination on both sides (back_70m +70m UV @ back), the locomotion speed decreases but remains active.

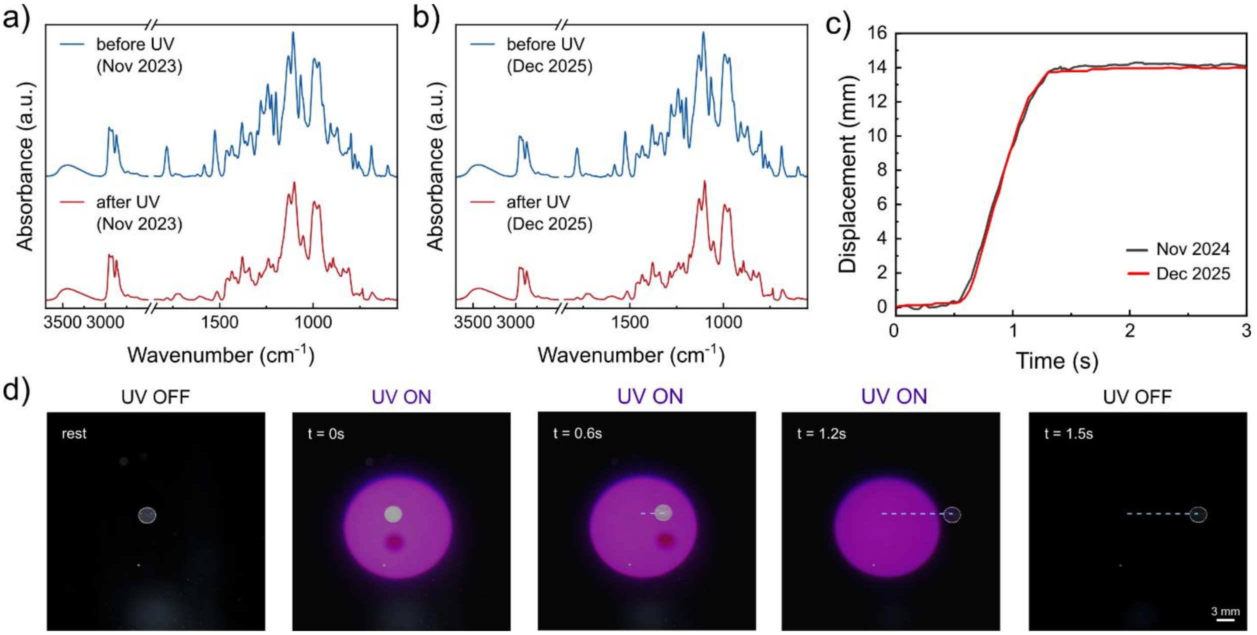

Figure S25 | Storage stability. FTIR absorption spectra of ONB-HFIP/PVB materials in **a)** November 2023 and the same materials in **b)** December 2025, both before and after UV exposure. **c)** Displacement as a function of time under UV exposure for fresh and for 1-year old samples, exhibiting the same locomotion behavior. **d)** Images of particle motion under UV illumination for 1-year old materials.

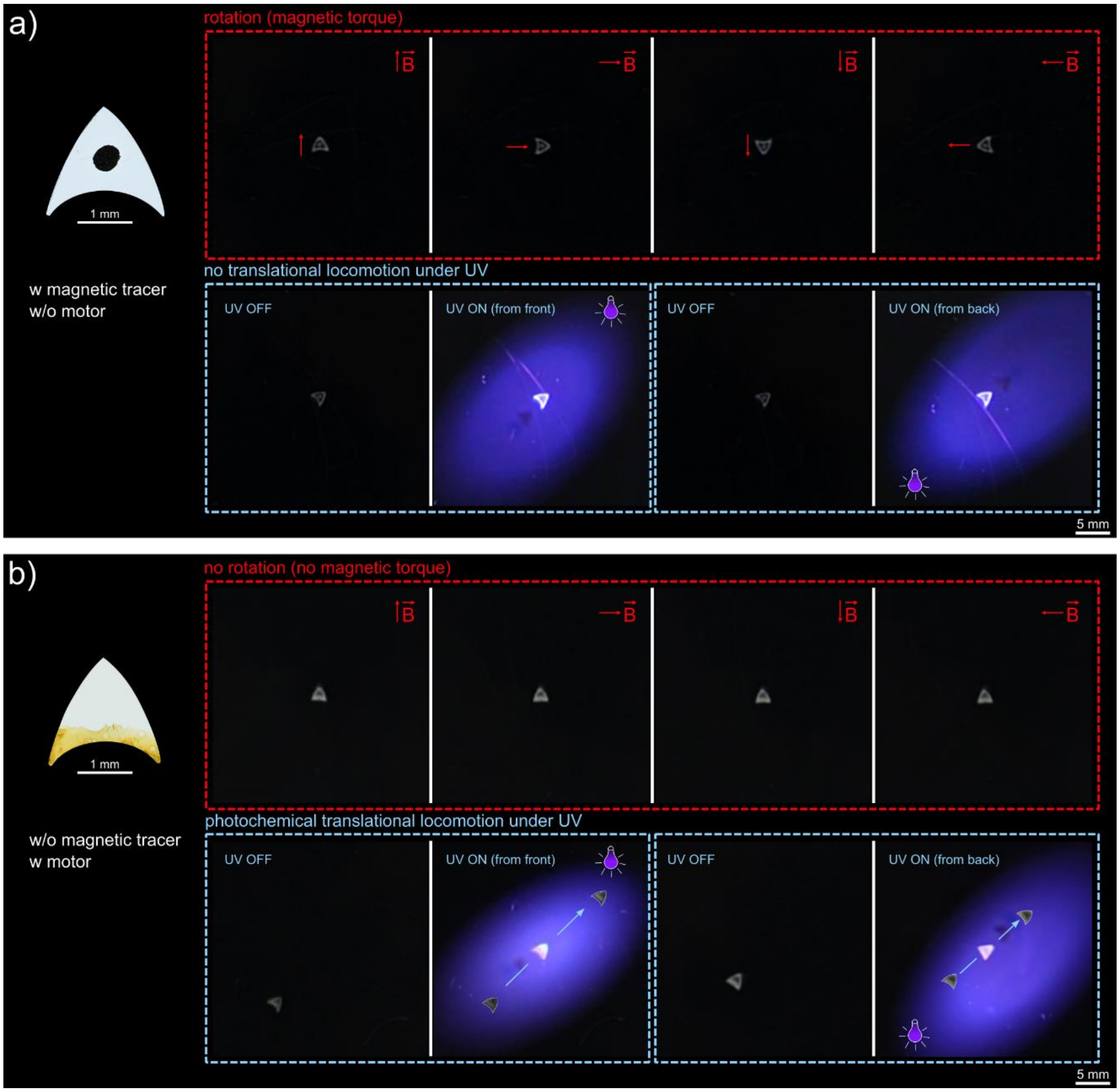

Figure S26 | Control experiments for hybrid swimming robots. **a)** Swimmers with only magnetic tracer, and without photochemical motor demonstrated only rotation via magnetic torque but no translation locomotion under UV illumination. **b)** Swimmers with only photochemical motor, and without magnetic tracer demonstrated only photochemical locomotion under UV (regardless UV illumination direction) but no magnetically driven rotation.

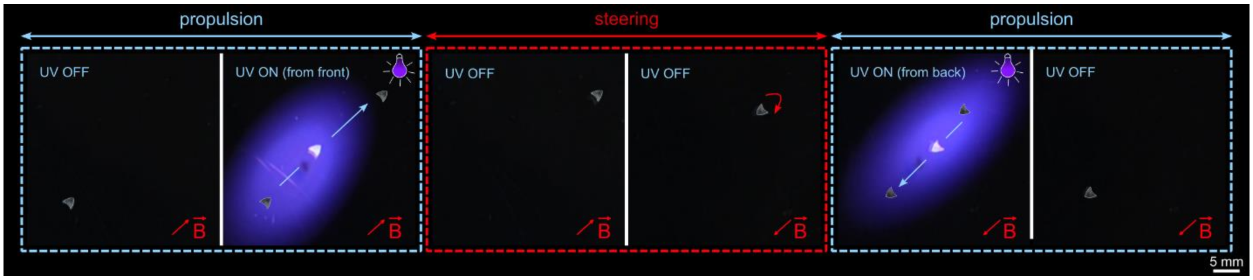

Figure S27 | Decoupled propulsion and steering of a hybrid swimming robot. Swimmers with both magnetic tracer and photochemical motor achieved orthogonal photo-driven propulsion and magnetic field-guided steering.

Supplementary Movies:

Movie S1 | Flow patterns during pumping tests

Movie S2 | Cyclic pumping tests

Movie S3 | Single particle pumping

Movie S4 | Particle cluster pumping

Movie S5 | Particle locomotion under localized UV illumination

Movie S6 | Hybrid swimming robots (control experiments)

Movie S7 | Locomotion trajectory control of hybrid swimming robots

Movie S8 | Navigation of a hydride swimming robot in confined environments
